# TOP2A inhibition and its cellular effects related to cell cycle checkpoint adaptation pathway

**DOI:** 10.1038/s41598-025-87895-8

**Published:** 2025-01-30

**Authors:** Maria Arroyo, M. A. Fernández-Mimbrera, E. Gollini, A. Esteve-Codina, A. Sánchez, Juan Alberto Marchal

**Affiliations:** 1 Departamento Biología Experimental, Universidad de Jaén, Paraje Las Lagunillas S/N E23071, Jaén, Spain; 2 Centre Nacional d’Anàlisi Genòmica (CNAG), Baldiri Reixac 4, 08028 Barcelona, Spain; 3 Universitat de Barcelona (UB), Barcelona, Spain; 4 Cell Biology and Epigenetics, Department of Biology, Technical University of Darmstadt, Darmstadt, Germany

**Keywords:** TOP2A inhibition, ICRF193, G2 checkpoint, Checkpoint adaptation, MCPH1, Microscopy, Checkpoints, Mitosis

## Abstract

In this study, we investigate the G2 checkpoint activated by chromosome entanglements, the so-called Decatenation Checkpoint (DC), which can be activated by TOP2A catalytic inhibition. Specifically, we focus on the spontaneous ability of cells to bypass or override this checkpoint, referred to as checkpoint adaptation. Some factors involved in adapting to this checkpoint are p53 and MCPH1. Using cellular models depleted of p53 or both p53 and MCPH1 in hTERT-RPE1 cells, we analyzed cell cycle dynamics and adaptation, segregation defects, apoptosis rate, and transcriptional changes related to prolonged exposure to TOP2A inhibitors. Our findings reveal that cell cycle dynamics are altered in MCPH1-depleted cells compared to control cells. We found that MCPH1 depletion can restore the robustness of the DC in a p53-negative background. Furthermore, this research highlights the differential effects of TOP2A poisons and catalytic inhibitors on cellular outcomes and transcriptional profiles. By examining the different mechanisms of TOP2A inhibition and their impact on cellular processes, this study contributes to a deeper understanding of the regulation and physiological implications of the DC and checkpoint adaptation in non-carcinogenic cell lines.

## Introduction

The topology acquired by chromatin at the end of G2 is a key factor for successful cell division. In this regard, physically entangled sister chromatids must be resolved to allow successful chromosome segregation^1^. TOP2A is the unique enzyme in eukaryotes able to remove the topological constraints between sister DNA helices established after DNA replication (a process known as decatenation). To achieve this, TOP2A forms a reversible covalent complex with DNA (cleavage complex) to promote the so-called Strand Passage Reaction (SPR), allowing passage of one intact DNA helix through a transient break induced in a second DNA helix^2^. The cleaved DNA molecule is rapidly re-ligated to achieve the untangling of the two intact chromatids. This is especially relevant for the proliferative division of cancer cells, where the inhibition of TOP2 can cause cancer cell death by inducing DNA damage^3^. Several agents targeting TOP2A have been used in clinical cancer therapy for over 30 years with great success^3^. These compounds are broadly divided into poisons and catalytic inhibitors^4^. TOP2A poisons (e.g., amasacrine, etoposide, doxorubicin) bind and arrest the covalent TOP2A-DNA complex with the DNA helix cleaved, resulting in a massive induction of DSBs throughout the genome. These drugs thus activate the well-established ATM kinase-dependent G2 checkpoint that responds to the presence of DSBs^5^. In contrast, TOP2A catalytic inhibitors are a heterogeneous group of compounds that do not stabilize the covalent enzyme DNA cleavage complex. Therefore, catalytic inhibitors do not produce DSBs directly. Interestingly, some catalytic inhibitors block ATP hydrolysis and trap TOP2A in its closed clamp conformation at the last stage of the SPR^6^. A distinct G2 checkpoint, named the decatenation checkpoint (DC), was identified in mammalian cells after the observation of G2 arrest produced by treatment with this type of catalytic inhibitors, for example, ICRF193^7–10^. The data compiled in recent years from several independent studies have clarified the biological importance of the DC as a TOP2A-dependent checkpoint response distinct from the G2 checkpoint pathway induced by DNA breaks^11–14^.

Several regulators are involved in the activation pathway (Table 1, glossary) of the TOP2A-dependent checkpoint. Of relevance, previous studies have demonstrated the requirement of p53 for a proficient TOP2A-dependent G2 arrest in response to ICRF193 treatment^14^. In hTERT-RPE1 cells and other non-cancer cell models, prolonged G2 arrest is triggered upon catalytic inhibition of TOP2A. In contrast, cancer cells frequently display a partial/minor G2 arrest upon treatment or even a complete loss of decatenation checkpoint activation. The latter might rely on p53 inactivation or loss-of-function in the carcinogenic state^1,14–16^. Therefore, the cellular capacity to avoid spontaneous bypass of the TOP2A-induced arrest, a phenomenon also known as “checkpoint adaptation” (Table 1, glossary), is a safeguard mechanism against cells entering mitosis with an excess of unresolved DNA catenanes. The lack of robustness of this checkpoint and the ability of cancer cells to bypass it leads to higher chromosome segregation errors, division failure, or frequent apoptosis. In our previous studies, we have shown that MCPH1 is a key regulator of the TOP2A-dependent G2 arrest^11,12,17^. MCPH1 is a gene mutated in primary microcephaly, a rare genetic syndrome characterized by pronounced reduction of the cerebral cortex and delayed growth^18^.

**Table 1:** Glossary of decatenation checkpoint-related events.

Term	Definition	References
Activation	Cell cycle arrest in G2 due to catalytic inhibition of TOP2A activity	^7–10^
Adaptation/bypass	Overriding the checkpoint blockage after a temporal cell cycle arrest and in the presence of the triggering agent	^11,12,17^
Recovery	Re-entering the cell cycle after the removal of the triggering agent/Exit from the arrest by extinguishing the checkpoint signal once the damage is dealt	^11–14^

MCPH1 function, a centrosomal protein that modulates chromosome condensation and cell cycle progression, is required to confer cellular adaptation to the DC, which means spontaneous checkpoint bypass after a transient G2 arrest induced by ICRF193. The pathway by which some cell types bypass the cell cycle arrest, i.e. induce checkpoint adaptation, and its consequences in the following cell divisions is still barely understood. Related to the DNA damage checkpoint, several studies are reporting the so-called checkpoint adaptation in different cell models. The latter corresponds to overriding the checkpoint imposed arrest after a temporal delay in G2, and subsequently entering mitosis with unrepaired DNA damage^19–22^. However, adaptation to the DC, meaning cells entering mitosis in the presence of DNA concatenations, has been barely studied. In lymphoblastoid cells derived from MCPH1 patients carrying loss-of-function MCPH1 mutations, bypass of the G2 arrest triggered by catalytic inhibitors of TOP2A was not observed^11,12,17^. In addition, it was shown that MCPH1 is not required to allow recovery (Table 1, glossary) from the DC, which happens upon removal of the triggering agent. This revealed that DC adaptation is a different process than DC recovery.

In this study, we provide further insight into the cellular consequences of prolonged activation and adaptation to the decatenation checkpoint in genetically stable cells. As a model, we have employed wild-type and genetically edited hTERT-RPE1 cells (MCPH1 Loss-of-Function (LOF)) to investigate the cell cycle dynamics, cell fate, and genome-wide transcriptional changes in response to prolonged incubation with TOP2A inhibitors.

## Results and discussion

### Characterization of MCPH1 loss-of-function (LOF) phenotype in RPE-1 genetically edited cells: prophase-like cells incidence, chromosome structure, and prolonged prometaphase

**Fig. 1:**
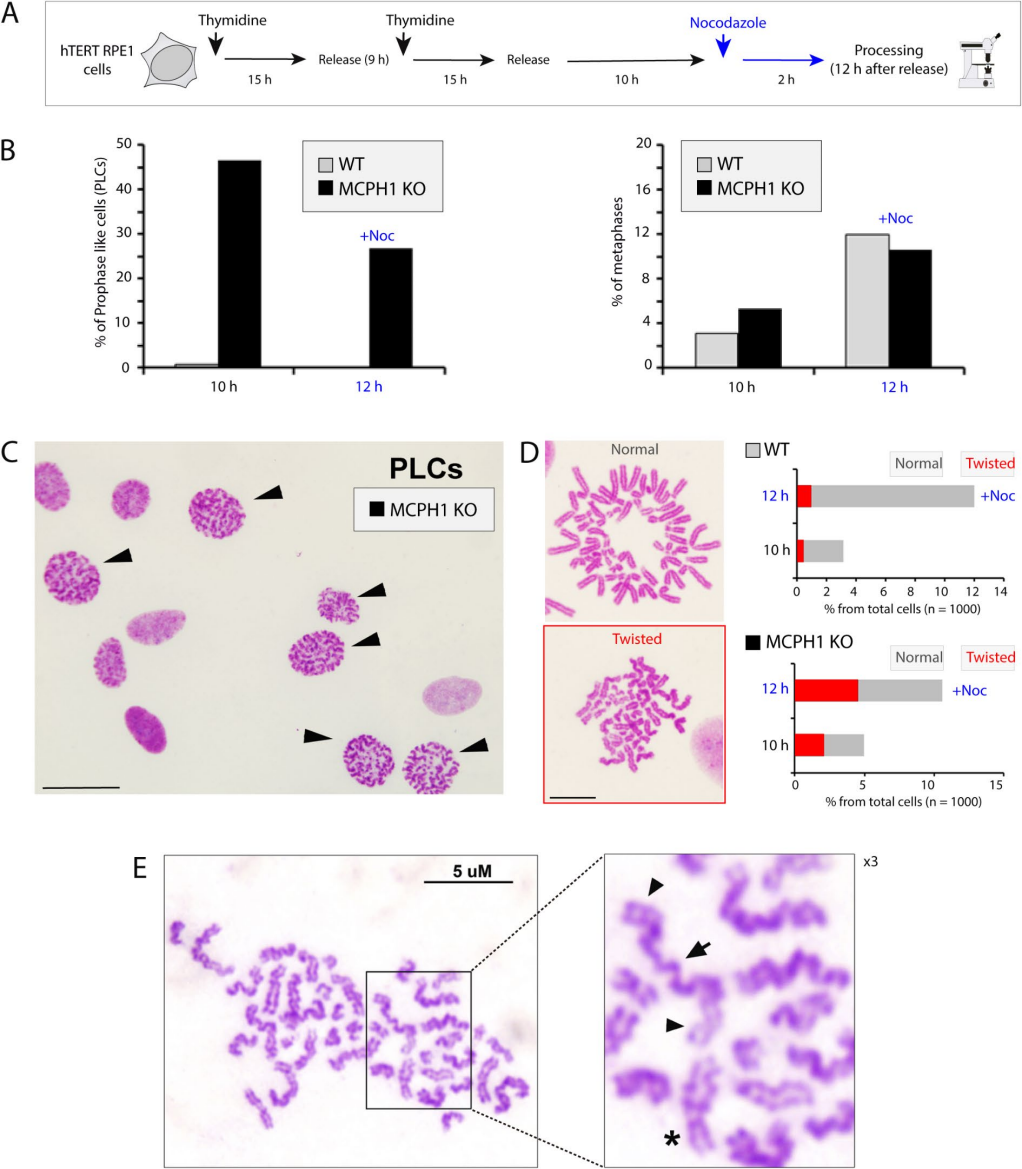
MCPH1 depletion in human RPE1 cells is associated with MCPH1 loss of function phenotypes: an increase in the fraction of prophase-like cells and twisted chromosomes. (**A**) Scheme illustrating the pipeline of the experiments, including synchronization, treatments, and processing times. Cells were synchronized at G1/S with a double thymidine block. Nocodazole was added at 10 h after the release of thymidine and cells were processed at 12 h. (**B**) Barplots showing the higher fraction of prophase-like cells (PLCs) in MPCH1 KO cells and a similar percent of metaphases in both WT and MCPH1 KO cell lines, with and without Nocodazole. (**C**) Representative microscopy images illustrating the PLC phenotype in MCPH1 KO cells. Cells showing condensed chromatin are marked with black arrows. Scale bar = 50 μm. (**D**) Representative images of metaphases with normal chromosome morphology versus condensed “twisted” or chromosomes with unresolved chromatids. The frequency of each metaphase type was quantified for each condition and is shown in the barplots on the left, with “twisted” fraction highlighted in red. Quantification showed a higher fraction of twisted chromosomes for MCPH1 KO cells. Scale bar = 5 μm. (**E**) An example of “twisted” metaphase in which the lack of resolution between sister chromatids is visible. Magnification × 3 on the right shows some examples of chromosomes with unresolved chromatids (black arrows) versus resolved chromatids (star). Two experimental replicates were performed and reproduced similar trends.

We first investigated the occurrence of the cellular and chromosome phenotypes associated with the lack of MCPH1 function in our cell model. As an MCPH1 mutated model, we made use of genetically edited RPE1 cells harboring a 7 bp deletion in exon 2, which results in a truncated MCPH1 protein (c.MCPH1del79_85; p.V13Gfs*33). The deletion was verified by sequencing and comparison with the reference genome (GRCh38, chromosome 8) and wild-type cells, all visualized using IGV (Integrative Genomics Viewer) (Fig. S1A). Western blot analyses of wild-type and edited cells confirmed the knockout (KO) of both MCPH1 isoforms (MCPH1 full length and short isoform lacking introns 9–14 (Δ9–14) at the protein level (Fig. S1B). MCPH1-depleted and RPE1 wild-type (WT) cells were also p53 defective (more details in “Material and methods”). In these cell lines, we investigated the occurrence of the Prophase-like cell (PLC) phenotype, a hallmark of MCPH1 deficiency that results in premature initiation of chromosome condensation at mid-G2 followed by delayed decondensation during the subsequent G1-phase^23,24^. The dynamics of PLCs during G2 progression were determined in cells released from S-arrest induced by thymidine block (Fig. 1A). The incidence of PLCs was significantly increased 10 h after release, a time window that overlaps with late G2, with 45% of total cells showing this phenotype (Fig. 1B-left). PLCs decreased 2 h later, which is expected since cells started to accumulate in mitosis at that time (nocodazole was added during the last 2 h to accumulate cells in mitosis). In WT cells the incidence of the PLC phenotype was negligible at late G2. Mitosis onset occurred at a similar rate in both WT and MCPH1 mutant cells as estimated from the fraction of metaphase cells observed at 10 and 12 h post-release (Fig. 1B-right) Representative images of PLCs are shown in Fig. 1C. These data align with previous studies that showed no significant differences in the rate of progression into mitosis of other cell types depleted of MCPH1 in comparison with controls, despite the occurrence of premature chromosome condensation^24^.

Next, we performed detailed cytogenetic analyses of chromosome morphology in MCPH1 mutant cells. As expected, we frequently observed hypercondensed wavy (“twisted”) chromosomes, a characteristic aberrant chromosome phenotype previously described in MCPH1 patient cells (Fig. 1D)^25^. The quantification of this “twisted” phenotype showed approximately a fourfold increase in MCPH1-depleted cells at 12 h post-release (Fig. 1D-left). These chromosomes did not present the expected rod-like shaped chromatids. Detailed examination revealed that sister chromatids were poorly resolved in some cases, and even varied in their resolution status along the same chromosome (Fig. 1E). These chromosome aberrations were barely observed in WT metaphase cells. Metaphases containing fully resolved sister chromatids were also observed in MCPH1 mutant cells. Therefore the process of chromatid resolution is delayed in MCPH1 mutant cells but might be completed in all cases before anaphase segregation. Of importance, in these metaphases, we did not observe an increase of chromosome structural aberrations such as chromatid or chromosome breaks or fusions in MCPH1 depleted cells compared with wild type (data not shown).

**Fig. 2:**
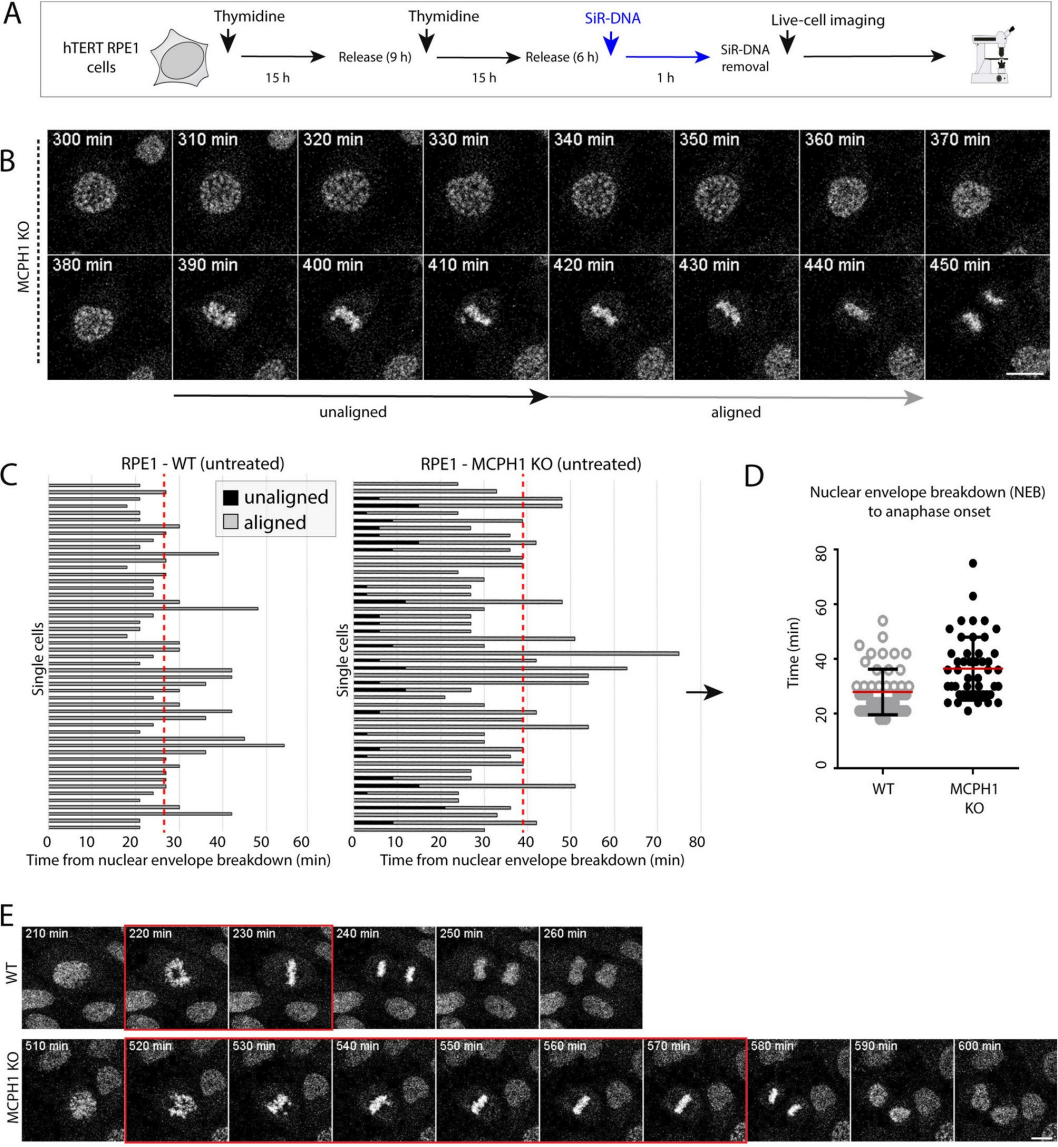
Prometaphase is elongated in RPE1 MCPH1-depleted cells due to delayed chromosomal alignment. (**A**) Scheme illustrating the pipeline of the experiments, including synchronization, treatments, and live-cell imaging. Cells were synchronized by a double thymidine block at the G1/S phase. DNA was stained for live cell imaging adding SiR-DNA at 6 h after release from thymidine. After 1 h of incubation with the DNA dye, new media was added to the cells for imaging. Images were acquired every 10 min. (**B**) Live-cell images illustrating premature chromosome condensation and extended prometaphase times for MCPH1 KO cells. Scale bar = 50 μm. (**C**) Single-cell quantification of chromosome alignment (aligned versus unaligned chromosomes) in live-cell time lapses. The time elapsed from nuclear envelope breakdown to anaphase onset (number of frames, 1 frame = 10 min) was quantified for each cell, and the average values for WT and MCPH1 KO cells are shown in (**D**) Red lines indicate the median, and whiskers indicate the standard deviation. On average, MCPH1 KO cells display longer chromosome alignment times. (**E**) Representative images of live-cell time lapses, showing different time points or frames for untreated WT and MCPH1 KO cells. Increased prometaphase time is visible for MCPH1-depleted cells compared with WT (frames highlighted with a red square). Scale bar = 50 μm. Two experimental replicates were performed and reproduced similar trends.

The occurrence of PLCs was also confirmed by live-cell imaging methods. To achieve this, cells were synchronized by double thymidine block and SiR-DNA was used to visualize chromosome condensation and segregation (see Fig. 2A). Of interest, in MCPH1 mutant cells, unaligned chromosomes were frequently observed in the subsequent frames after nuclear envelope breakdown (Fig. 2B). Moreover, when analyzing the number of frames required to align chromosomes and reach the onset of anaphase for each live-cell (Fig. 2C), we found that the average time required to complete full metaphase alignment and start anaphase was increased compared with WT cells (Fig. 2D,E). These data confirm that chromosome biorientation during mitosis is compromised in MCPH1 mutant RPE1 cells during mitotic division, in consonance with previous reports in other cell models^17,24^. It is tentative to speculate that delayed/prolonged sister chromatid resolution might be functionally related to the increased duration of chromosome alignment in the metaphase plate. Furthermore, the data show that the prolonged prometaphase phenotype is conserved between different cell types and is not cancer-cell specific. Further mechanistic analyses are required to confirm the proposed interplay between sister chromatid resolution and the duration of chromosome alignment.

In summary, the RPE1 MCPH1 mutant cell line recapitulates the described MCPH1-loss-of-function phenotypes in unperturbed cell cycle conditions. This diploid, genetically stable, and non-transformed cell line is a bona fide MCPH1 LOF cell model to further investigate cell cycle dynamics and transcriptional profiles upon prolonged catalytic inhibition of TOP2A.

### MCPH1 is required to achieve a permanent G2 arrest after high-dose inhibition of TOP2A in p53-deficient cells

Recent studies have demonstrated the importance of p53 status for the long-term robustness of the ICRF193-induced G2 arrest. Thus, in hTERT-RPE-1 cells with functional p53 a complete G2 arrest has been described in response to catalytic inhibition of TOP2A with ICRF193^14^. Therefore, to gain further insights into the nature of this response we decided to investigate the contribution of MCPH1 as a regulator of the ICRF193-related checkpoint robustness. Previous studies showed that MCPH1 function is required for the spontaneous bypass of the decatenation checkpoint, i.e. overriding the checkpoint after a temporal G2 arrest despite the persistence of the TOP2A inhibitor^11,17^. These studies were performed in non-immortalized lymphoblastoid cell lines and carcinogenic HeLa cells. This cell response is also known as checkpoint adaptation, which is conceptually different from checkpoint recovery in which the cells can continue through the cell cycle after removal of the stress agent, in this case, TOP2A inhibitors^26^.

**Fig. 3:**
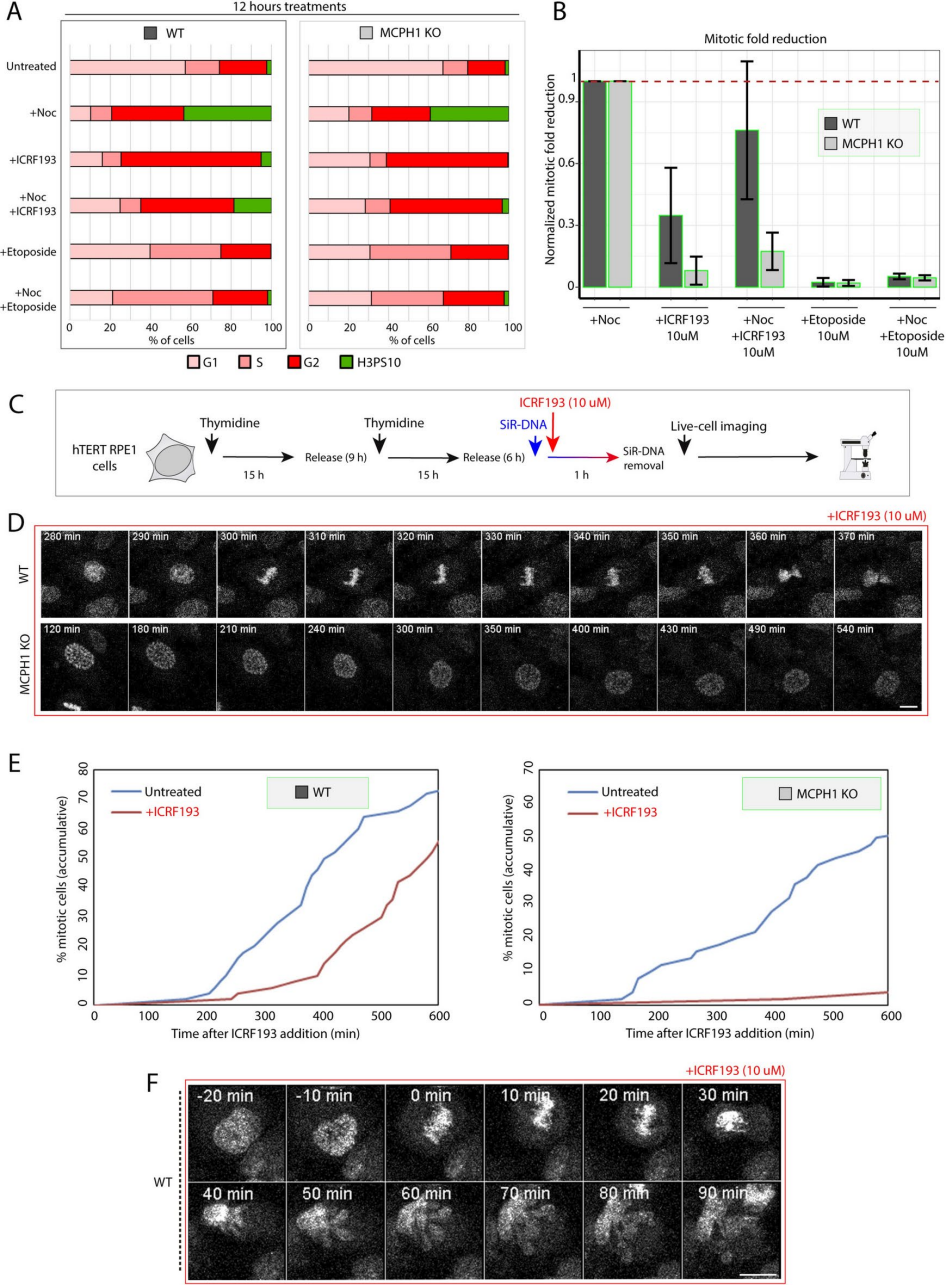
Permanent G2 arrest upon catalytic TOP2A inhibition is not achieved in MCPH1-depleted cells. (**A**) Barplots showing the fraction of cells in different cell cycle substages obtained by flow cytometry analysis (G1, S, G2, and H3PS10 or mitotic cells in green). The latter is comparatively shown for WT and MCPH1 KO cells subjected to different treatments: control (untreated), + Noc (Nocodazole), + Noc + ICRF193 (TOP2A catalytic inhibitor), + Etoposide (TOP2A poison), + Noc + Etoposide. The reduction in the fraction of mitotic cells upon these treatments is shown in (**B**), WT is shown in dark grey, and MCPH1 KO in light grey. A higher reduction in the fraction of mitotic cells is shown for MCPH1 KO cells compared with WT for ICRF193 treatments, while a similar low fraction of mitosis was found for etoposide treatments. (**C**) Scheme illustrating the pipeline of the experiments, including synchronization, treatments, and live-cell imaging. Cells were synchronized by double thymidine blocks. 6 h after release from thymidine, DNA staining SiR-DNA and ICRF193 were added. After the removal of SiR-DNA and 1 h ICRF193 incubation, live-cell images were acquired every 10 min. (**D**) Representative images of live-cell time-lapse tracking during ICRF193 incubation, showing WT cells entering mitosis and displaying segregation defects, while MCPH1 KO cells stay arrested in G2. Scale bar = 50 μm. (**E**) Line plots showing the quantification of mitotic cell accumulation in untreated (blue) versus ICRF193 treatment (red). WT cells are shown on the left and MCPH1 KO cells are on the right. This quantification was performed in the live cell movies shown in (**D**). (**F**) Live-cell representative images of WT cells treated with ICRF193 bypassing G2 arrest and entering mitosis without proper chromosome segregation. Scale bar = 50 μm. Two experimental replicates were performed and reproduced similar trends.

First, we analyzed the cell cycle profile by flow cytometry in unsynchronized cells incubated with ICRF193 for 12 h. To assess the dynamics of G2/M transition, nocodazole was also added alone or combined with ICRF193 to trap those cells entering mitosis, which were detected by flow cytometry using histone H3PS10 as a mitotic marker. Furthermore, we compared the cell cycle dynamics after treatment with a TOP2A poison (etoposide), which induces DSBs and activates the DNA damage checkpoint^5^. The results are presented in Fig. 3. In RPE1 WT cells treated with ICRF193, there was an increase in the G2 fraction (and a reduction of G1) compared with untreated cells (Fig. 3A, Fig. S2A). Figure S2A shows the histograms of propidium iodide (PI) profiles obtained from flow cytometry experiments for wild-type (WT) after 12 h of treatments under different conditions (Untreated, + Nocodazole (Noc), + ICRF193, + Noc + ICRF193, + Etoposide, and + Noc + Etoposide). Despite this accumulation, cells were able to progress into mitosis as the mitotic index of cells treated with ICRF193 and nocodazole for 12 h was reduced only to half compared with cells single treated with nocodazole for the same period (Fig. 3A,B). Therefore, cells retain the capacity to spontaneously bypass the checkpoint triggered by ICRF193 after some temporal arrest in G2. These dynamics coincided and were expected according to^14^ for p53 mutant RPE1 cells with functional MCPH1. When the same analyses were performed in RPE1 MCPH1 mutant cells, the accumulation of mitotic cells was extremely reduced upon treatment with ICRF193 alone or combined with nocodazole for 12 h. The fold reduction in the mitotic index compared to cells single-treated with nocodazole for the same period dropped significantly (Fig. 3B). As a consequence of MCPH1 being mutated, RPE1 cells with defective p53 are permanently arrested in G2 after 12 h of incubation with ICRF193 (Fig. 3A and Fig. S2B) and could not spontaneously bypass the checkpoint. Figure S2B shows the histograms of propidium iodide (PI) profiles obtained from flow cytometry experiments for MCPH1 depleted cells upon 12 h of treatments under different conditions (Untreated, + Nocodazole (Noc), + ICRF193, + Noc + ICRF193, + Etoposide, and + Noc + Etoposide) and in comparison with WT cells ( Fig. S2B). Therefore, the temporal nature of the ICRF193-induced G2 arrest became nearly permanent in p53 mutant RPE1 cells if MCPH1 function was also lacking. This result demonstrates that MCPH1 regulates the ICRF193 response in both tumor and non-tumor cell contexts and that MCPH1 depletion restores the robustness of the decatenation checkpoint arrest in the absence of p53.

When the same analyses were repeated using etoposide instead of ICRF193, both RPE1 WT and MCPH1 cells showed similar cell cycle dynamics. In this case, after 12 h of incubation, an evident S-phase arrest was triggered which was also accompanied by a minor G2 arrest (Fig. 3A and Fig. S2A,B). This is probably a consequence of the massive occurrence of DSBs in the DNA that interfere with the replication process^21,27–29^. In this extreme DNA damage context, cells stopped entering mitosis; thus the mitotic index was near zero in all etoposide-treated samples (Fig. 3B). The latter demonstrates the specificity of the cell cycle response by decatenation checkpoint activation after ICRF193 treatment, involving regulation by MCPH1. This pathway is distinct from the signaling activated upon etoposide treatment, related to double-strand breaks (DSBs) damage.

We next used confocal live-cell imaging to investigate in detail the cell cycle dynamic in these cells and infer the timing of response to ICRF193 (Fig. 3C). As depicted in Fig. 3D,E, WT cells treated with ICRF193 arrested transiently in G2, and after approximately three hours began to spontaneously adapt to the checkpoint and progressed into mitosis (revealed by the occurrence of nuclear envelope breakdown) (Fig. 3D-upper panel). The latter was quantified in live-cell movies by the cumulative fraction of mitotic cells over the time course of the experiments. However, the cell cycle dynamics described for WT were not observed in RPE1 MCPH1 KO cells. On the contrary, MCPH1-depleted cells treated with ICRF193 did not progress into mitosis and remained as PLCs during the full-time course of the experiment (i.e. 10 h) (Fig. 3D-button panel). Interestingly, we observed different aberrant phenotypes in WT cells bypassing the decatenation checkpoint and progressing into mitosis. These defects appeared as segregation problems and atypical anaphases that were unable to separate into two independent nuclei, generating a multilobulated amorphous nucleus (Fig. 3F). These non-segregated nuclei after completion of anaphase are a direct consequence of bypassing the checkpoint and possessing a high number of DNA concatenations that are incompatible with mitotic division. Whether this population of cells can continue or not with subsequent cell cycles or have a higher apoptosis rate and/or genomic instability needs to be further investigated.

### Cellular defects associated with decatenation checkpoint bypass: segregation defects and ultra-fine bridges (UFBs)

**Fig. 4:**
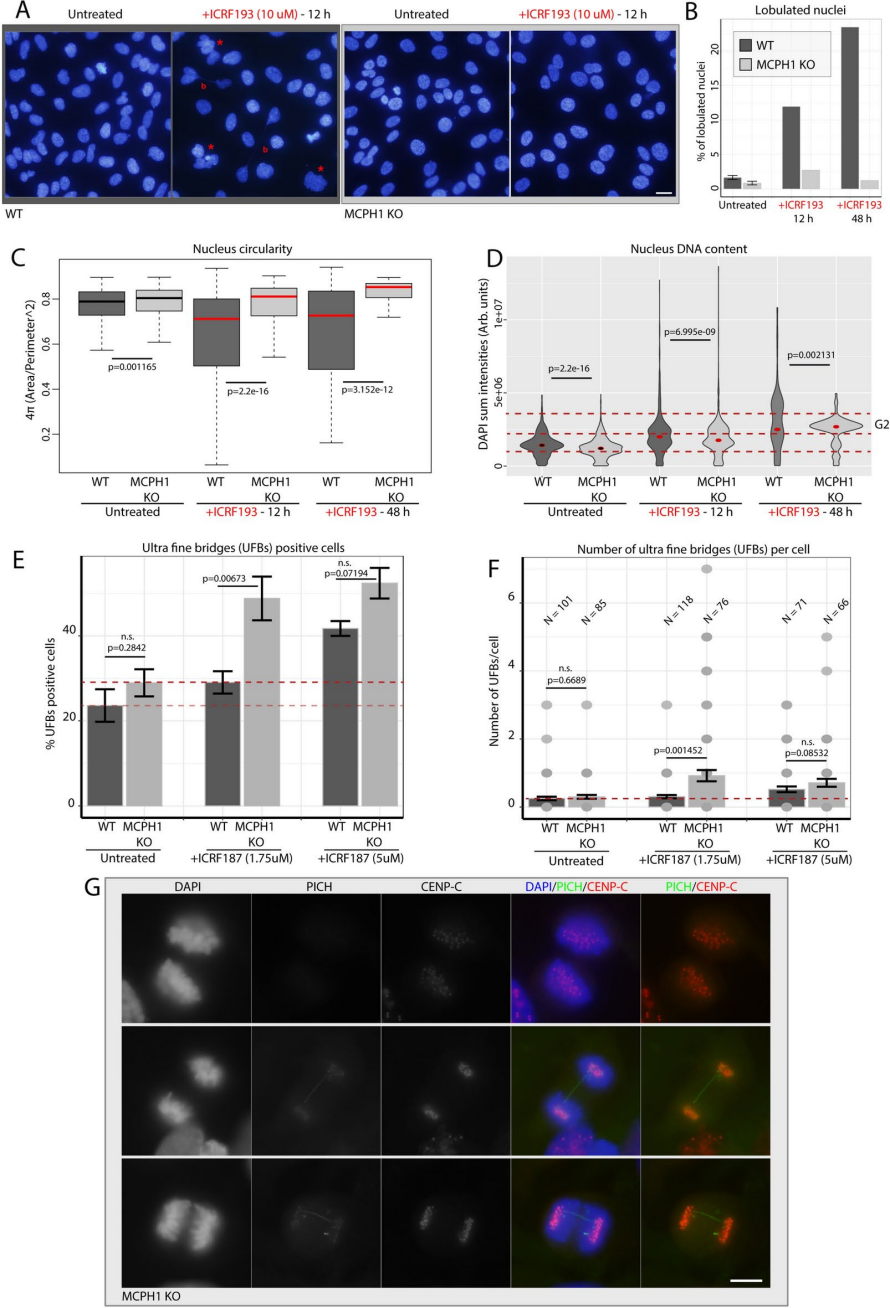
Cellular defects associated with bypassing the G2 decatenation checkpoint. (**A**) Representative microscopy images of nuclei stained with DAPI in WT and MCPH1 KO cells with and without ICRF196 treatment (12 h). The effect of the incubation with ICRF193 nuclei shape and morphology can be visualized predominantly for WT cells (examples highlighted with red stars). Segregation defects like DNA bridges are indicated with red “b”. Scale bar = 50 μm. (**B**) Fraction of nuclei showing multilobulated appearance in WT and MCPH1 KO cells under different treatments, showing an increase for WT cells (dark grey) treated with ICRF193. (**C**) Quantification of nuclear shape by measurements of circularity using FIJI. Nuclei were segmented using the plugin “StarDist” and values of circularity for each cell were plotted. WT cells treated with ICRF193 showed a decrease in nucleus circularity values. Values of DAPI sum intensity were plotted as violin plots and are shown in (**D**), where the dashed red line indicates the approximate range of DAPI sum intensity values corresponding with G2 DNA amount, showing the highest fraction of G2 cells for MCPH1 KO cells treated with ICRF193 for 48 h. N (cells) = 1654–113. These quantifications were performed in the data sets from (**A**) and (**B**). (**E**) Fraction of cells positive for ultra-fine bridges (UFBs), based on PICH (Plk-interacting checkpoint helicase) signal (N (cells) = 119–67). UFBs show a higher increase for MCPH1 KO cells treated with ICRF187. The number of UFBs per cell is shown in the barplot of (**F**), and representative microscopy images of this immunofluorescence, with examples of UFBs, are shown in (**G**). Scale bar = 20 μm. Independent two-group comparison was made with the Wilcoxon-Mann–Whitney test, and p-values are given in the plots between the top of two samples subjected to comparison (n.s. = not significant is given for p-values > or equal to 0.05). Two replicates were performed. The cells analyzed showed the reported behavior of the representative images selected.

We next examined in detail mitotic progression in cells that bypassed the ICRF193-induced G2 arrest. To achieve this, we analyzed lobulated nuclei or mitotic figures in fixed cells after the treatment with ICRF193 for 12 or 48 h. To visualize the DNA and the morphology of the nucleus, cells were stained with DAPI. As shown in Fig. 4A, for WT cells treated with ICRF193 we frequently observed persistent long DNA stretches connecting segregated cells (marked as a red “b”) as well as multilobulated amorphous nuclei (marked with a star). After quantification, we found approximately a tenfold increase in the fraction of lobulated nuclei in WT cells treated with ICRF193 for 12 h, and a 20-fold increase after 48 h, compared with untreated WT cells or MCPH1 depleted cells (Fig. 4B). For the latter, the fraction of lobulated nuclei remained similar to untreated cells. Furthermore, we analyzed other nuclear shape descriptors like circularity. In line with the higher fraction of lobulated cells in WT cells after ICRF193 treatments, nuclear circularity (defined as 4π (Area/Perimeter^2^)) was also significantly reduced in these samples (Fig. 4C). In addition, we analyzed DNA content in WT and MCPH1 depleted cells after 12 and 48 h of ICRF193 treatment, using DAPI sum intensity as a proxy for DNA content. Violin plots showing the distribution of DAPI sum intensity values for each condition are shown in Fig. 4D. Interestingly, after 48 h of treatment, the accumulation of cells arrested in G2 is much higher for MCPH1-depleted cells, while the distribution of DAPI sum intensity values for WT cells incubated with ICRF193 reached much higher levels (Fig. 4D), suggesting the occurrence of endomitosis events and associated genomic instability. These lobulated nuclei after inhibition of TOP2A catalytic activity represent variations of the so-called “cut phenotype”, that result from exiting mitosis without segregating the chromosomes and then entering a new S-phase^30^. The phenotype observed in fixed cells coincides with what we observed in WT cells tracked by live-cell imaging, bypass of the ICRF193-induced G2 arrest, and aberrant segregations (see Fig. 3F). MCPH1-depleted cells do not spontaneously bypass the arrest induced by ICRF193, therefore this phenomenon is very rare.

Regarding the higher occurrence of DNA bridges mentioned in Fig. 4A, previous studies in HeLa cells depleted of MCPH1 function also showed an increase in the basal level of these major anaphase errors: laggards and bridges. The incidence of those errors however occurred at similar rates to control cells when the decatenation capacity was compromised by using mild conditions for TOP2A inhibition^17^. Therefore, we studied this phenomenon using mild conditions of TOP2A inhibition. Those mild conditions, using low concentrations of ICRF187, are necessary to avoid a permanent G2 arrest in MCPH1 mutated cells to investigate the incidence of anaphase errors in both WT and MCPH1 depleted cells. In this study, we did not observe major anaphase errors in RPE1 cells, either WT or MCPH1-depleted without ICRF (unperturbed cell cycle conditions)(data not shown).

However, minor failures in resolving entangled sister chromatids might result in ultrafine bridges (UFBs), a type of error that cannot be visualized with conventional DNA dyes and requires special detection with specific antibodies against PICH (Plk1-interacting checkpoint helicase), a protein that decorates UFBs^31,32^. Therefore, we examined the incidence of UFBs in RPE1 WT and MCPH1 mutant cells during anaphase after incubation for three hours with different concentrations of ICRF187 (mild dose_5 uM, and low dose_1.75 uM) (Fig. 4E–G). Although ICRF187 is a less potent TOP2A catalytic inhibitor than ICRF193, it still induces a G2 arrest at high concentrations in MCPH1-depleted cells, similar to ICRF193^17^. The timing and duration of the incubation used allow us to score anaphase cells that maintained full TOP2A activity during replication. This is of interest to analyze the decatenation process of already fully replicated chromatids (and avoid UFBs that arise as a consequence of incompletely replicated regions/loci). After performing immunofluorescence against PICH in cells treated with ICRF187 as described before, we observed a higher rate of UFBs in MCPH1-depleted cells compared with WT. This higher incidence of UFBs, corresponding to nearly a twofold increase, was observed with the low dose of ICRF187, while for WT cells the incidence was similar to untreated conditions (Fig. 4E,F). Representative images of these UFBs, including CENP-C to visualize the centromeres, are shown in Fig. 4G. Interestingly, these differences in the fraction of UFBs between WT and MCPH1-depleted cells were no longer observed at higher ICRF187 concentrations (5 uM) (Fig. 4F). These results suggest that in RPE1 cells lacking MCPH1 function, the removal of DNA catenations between sister chromatids is already challenged by minor perturbations of TOP2A catalytic activity. One tentative idea is that the longer times with hypercondensed chromatin during G2, the common phenotype after MCPH1 depletion, results in an excess of DNA catenation. Such catenation excess would require optimal chromatin disentanglement conditions, e.g. fully active TOP2A. Supporting this hypothesis is another phenotype associated with MCPH1’s lack of function, frequently unresolved “twisted” chromosomes in MCPH1 mutated cells^25^. Of interest, a recent study revealed the key role of histone modifications H3K27me3 and H4K20me3 for the decatenation activity of TOP2A. The chromatin tether domain of TOP2A interacts with methylated nucleosomes, which is crucial for high-fidelity chromosome segregation^33^. Due to the interplay of epigenetic marks and chromatin condensation, it would be of interest to further explore the impact of such histone modifications on the permanent ICRF-induced G2 arrest observed in MCPH1-depleted cells.

### Consequences of long-term incubation with TOP2A inhibitors on cell cycle dynamics and cellular apoptosis

**Fig. 5:**
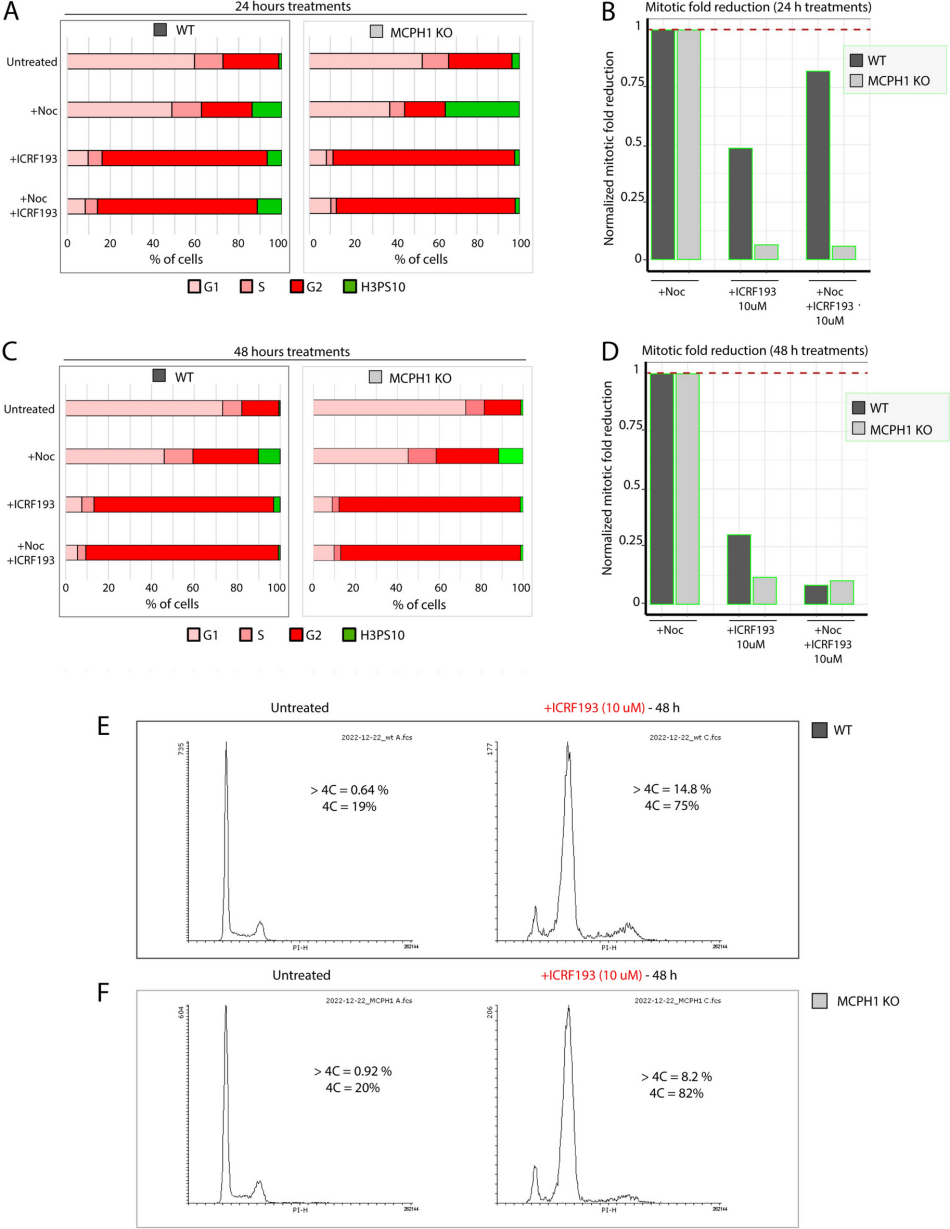
Long-term incubation effects of TOP2A inhibition in cell cycle dynamics. (**A**) Barplots showing the fraction of cells in different cell cycle substages obtained by flow cytometry analysis (G1, S, G2, and H3PS10 or mitotic cells). The latter is comparatively shown for WT (left) and MCPH1 KO (right) cells subjected to different treatments during 24 h (Control = untreated, + Nocodazole (+ Noc), + ICRF193 (TOP2A inhibition), + Noc + ICRF193). The reduction in the fraction of mitotic cells (green, H3PS10) upon these treatments is shown in (**B**), compared with cells treated only with + Noc. WT is shown in dark grey, and MCPH1 KO is shown in light grey. The fraction of mitotic cells is much lower in MCPH1 KO cells. The same experimental approach was performed, increasing the treatment length to 48 h. Barplots for 48-h treatments are shown in (**C**), and the reduction in the fraction of mitotic cells after 48 h is shown in (**D**). For (**B**) and (**D**), dashed red lines highlight the mitotic fraction in cells treated with + Noc. The latter corresponds to 1 since these samples were used for normalization. (**E**) and (**F**) show histograms of flow cytometry propidium iodide profiles of the cell population for each treatment: Untreated and 48 h with ICRF193, indicating the fraction of cells with 4C and > 4C DNA content, both increased in the treatment with ICRF193. Propidium iodide profiles for 24-h incubation can be found in Fig. S3. Two experimental replicates were performed and reproduced similar trends.

Next, we investigated the effects of prolonged treatment with a high dose of ICRF193 on cell cycle dynamics. To do that, we investigated the cell cycle profile after 24 and 48 h of incubation with ICRF193 by FACS (Fig. S3). Figure S3A shows the histograms of propidium iodide (PI) profiles obtained from flow cytometry experiments for wild-type (WT) cells after 24 or 48 h under different conditions (Untreated, + ICRF193), and compared with MCPH1 KO cells shown in Fig. S3B. The fraction of cells with 4C DNA content for each cell line and condition is shown. We aimed to infer whether the differences in the adaptation ability of MCPH1 depleted and wild-type cells to the G2 arrest induced by ICRF193 observed after 12 h are still maintained or, on the contrary, if both cell types respond similarly with long-term TOP2A inhibiting conditions. These data are presented in Fig. 5 and Fig. S3. After 24 h of treatment with ICRF193 and nocodazole, MCPH1 mutated cells show an extreme reduction in the fraction of mitotic cells compared with cells treated only with nocodazole. However, in WT cells the reduction is less pronounced (Fig. 5A,B). The differences between both cell types are less striking when these same treatments are maintained for 48 h (Fig. 5C,D). In this case, both MCPH1 depleted and WT cells show a drastic reduction in the fraction of mitotic cells. In parallel, this is accompanied by a high increase in the fraction of G2 cells (revealed by a 4C DNA content) (Fig. 5C,E,F). These data indicate that differences in the adaptation capacity are still observed between both cell types after 24 h of catalytic inhibition of TOP2A, being progressively reduced when incubation time is extended. It was noticeable that the accumulation of cells with > 4C DNA (8C) content after 48 h of treatment with ICRF193 (see propidium iodide (PI) profiles in Fig. 5E,F and Fig. S3) was significantly increased in WT cells compared with MCPH1 KO. This observation agrees with the results shown in Fig. 4, showing a high incidence of amorphous lobulated nuclei in WT cells after 48 h of ICRF193 incubation, which is barely observed in MCPH1 KO. Overall, this suggests that WT cells are more prone to endo-replication due to progressing through the next cell cycle in the absence of chromosome division as a consequence of ICRF193 being present.

**Fig. 6:**
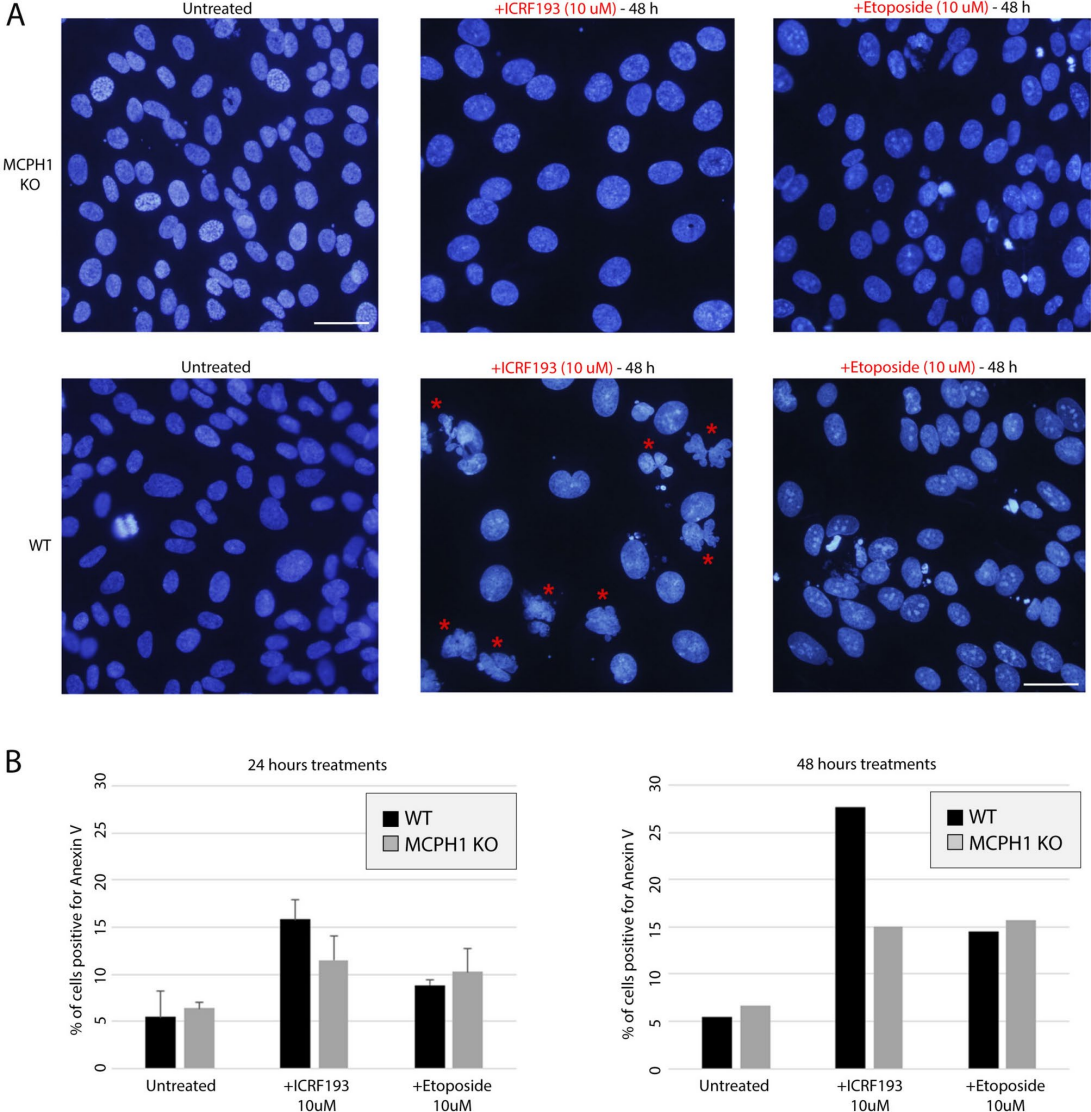
MCPH1-depleted cells showed a reduced apoptosis rate after long-term TOP2A inhibition. (**A**) Representative microscopy images of nuclei stained with DAPI in WT and MCPH1 KO cells, with and without ICRF196 or Etoposide treatment (48 h). The effect of the incubation with ICRF193 nuclei shape and morphology can be visualized predominantly for WT cells (examples highlighted with red stars). Scale bar = 100 μm. (**B**) Flow cytometry analysis to quantify the fraction of cells positive for Anexin V as a marker for apoptosis. Cells subjected to the long-term treatments shown in (**A**) (Untreated, + ICRF193, and Etoposide), were analyzed by flow cytometry. Barplots show the fraction of apoptotic cells (Anexin V positive) for these treatments, at 24 h (left) and at 48 h (right). WT cells treated with ICRF193 showed a higher apoptosis rate compared with MCPH1 KO cells (not entering mitosis, Fig. 5), showing even larger differences upon 48 h treatments. Two experimental replicates were performed and reproduced similar trends.

Multilobulated phenotypes are shown in Figs. 3F, 4A, and 6A, likely represent variations of the so-called “cut phenotype”, resulting from exiting mitosis and entering a new cell cycle without segregating the chromosomes as a consequence of the inhibition of TOP2A catalytic activity^30^. It is important to highlight that these aberrant cells exit mitosis with a DNA content of 4C since chromosome segregation was disrupted. Consequently, within the first cell cycle affected by TOP2A inhibition, they would be scored as G2 in the FACS profiles according to their DNA content, complicating the interpretation of the data. For this reason, the combined evidence presented here (live-cell imaging, fixed cells, and cell nuclei morphology) provides a better understanding and reveals that cell cycle progression was not interrupted at G2 during prolonged incubation with ICRF193 in all RPE-1 WT cells. Thus, it is crucial to combine both flow cytometry and microscopy techniques to fully understand the cell cycle dynamics and the robustness of cell cycle checkpoints together with effects on cell morphology, and/or chromatin.

As we have shown in this study, WT cells were able to progress through mitosis and enter into a new cell cycle despite the incapacity to segregate the chromosomes. Due to these findings, we decided to investigate whether prolonged incubation with ICRF193 during 24 or 48 h results in increased apoptotic cell death. Cells were treated as described before, and the incidence of lobulated nuclei was observed again almost exclusively for WT cells treated with ICRF193 for 48 h. The multilobulated phenotype was almost absent in the other conditions, including untreated, etoposide, as well as in MCPH1-depleted cells (Fig. 6A). The same samples used for DAPI staining were measured by flow cytometry to infer the frequency of cells targeted by Anexin V as a marker for apoptotic cells (Fig. 6B). This analysis revealed an increase in the fraction of Anexin V positive cells after TOP2A inhibition, and a slight increase in the fraction of WT cells positive for Anexin V compared with MCPH1 KO after 24 h of incubation with ICRF193. This difference is magnified at 48 h. When the same analyses were performed in etoposide-treated cells, no differences were observed between the cell lines. Our analyses thus suggest that in wild-type cells prolonged incubation with ICRF193 induces a higher rate of apoptosis compared with MCPH1 mutated cells. Since in wild-type cells, apoptotic death events are accompanied by mitotic cell division in the absence of successful chromosome segregation, (due to lack of TOP2A catalytic activity), it is tentative to speculate that both phenotypes are mechanistically linked. In the case of MCPH1 mutated cells, their permanent G2 arrested status does not result in an apoptotic response.

### Wide transcriptional profile analysis upon cellular response to ICRF193-induced G2 arrest

Finally, we aimed to characterize transcriptional changes and/or affected pathways in response to the presence of TOP2A closed-clamp complexes in the chromatin, an aspect that has been less investigated^1^. Of particular interest was to identify the genes and pathways showing expression changes upon prolonged catalytic inhibition of TOP2A with ICRF193. In addition, we aimed to identify transcriptional changes that may be specific to the checkpoint adaptation response, screening for differences between WT cells, showing checkpoint adaptation, and MCPH1 depleted cells, lacking such capacity. Therefore, RPE-WT and MCPH1 KO cells were subjected to prolonged incubation with ICFR193 (concentration 10 uM) for 12 h. Each cell sample, including untreated cells, was divided into two aliquots: one being processed for FACS analysis to obtain cell cycle profiles and the other for RNAseq analysis. FACS cell cycle profiles were similar to those presented in Fig. 3A and Fig. S2, confirming the different cell cycle dynamics of WT and MCPH1-depleted cells upon prolonged inhibition of TOP2A catalytic activity.

**Fig. 7:**
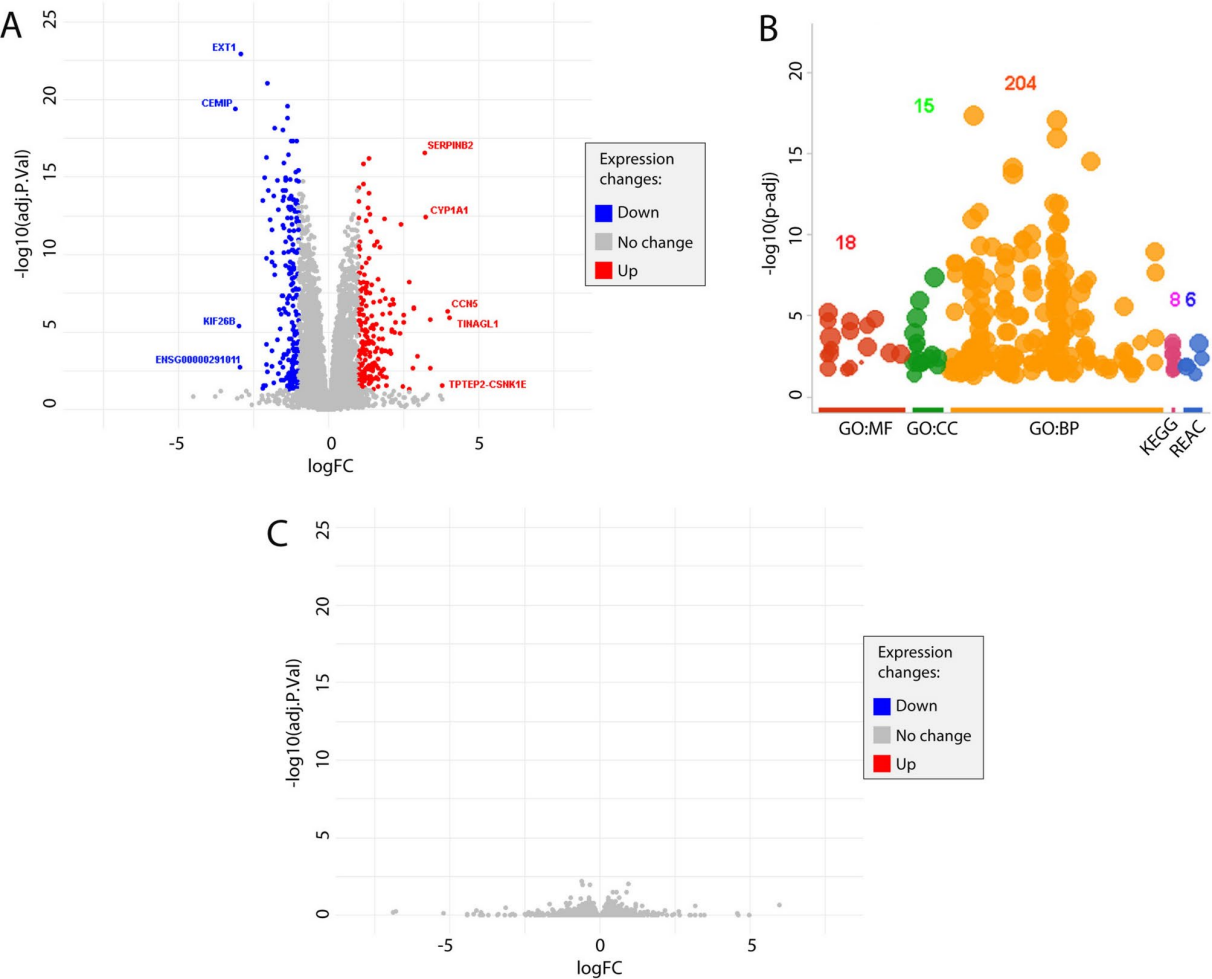
RNA-seq analysis of transcriptional response to catalytic inhibition of TOP2A. (**A**) Analysis of transcription by pairwise comparison and principal component analysis (PCA). Volcano plots showing DESeq2 for gene expression, with 207 genes downregulated (fold change ≤ 0.5) and 197 genes upregulated (fold change ≥ 2) (see p-values in Table S1). The most significant downregulated genes are shown in blue (CEMIP (− 3.10 log2FC), KIF26B (− 2.98 log2FC), and EXT1 (− 2.92 log2FC)). The most significant upregulated genes are shown in red (TINAGL1 (4.03 log2FC), CCN5 (3.97 log2FC), and TPTEP2-CSNK1E (3.78 log2FC)). Genes with no transcriptional changes are shown in grey. (**B**) Functional enrichment analyses for differentially expressed genes (absolute fold change ≥ 2). Each color in the plot corresponds with gene ontology (GO) database: red for “GO:MF” (molecular function), green for “GO:CC” (cell cycle), orange for “GO:BP” (biological processes), pink for “KEGG” (Kyoto Encyclopedia of Genes and Genomes), and blue for “REAC” (Reactome). See Table S3 for a full description. The list of terms with their corresponding p-values, intersection size, and enrichment ratio, can be found in Table S2. (C) Volcano plot showing the comparison of the transcriptional profile of RPE1 WT cells upon prolonged ICRF193 incubation with the one observed in RPE1 MCPH1 KO cells after the same treatment. DEG data of both samples were normalized against each untreated counterpart, filtering conditions: adj.p.value ≤ 0.05 and absolute fold change > 2). Genes with no transcriptional changes are shown in grey. Three experimental replicates were performed.

First, we analyzed the transcriptional pathways altered upon prolonged stress by catalytic inhibition of TOP2A. We performed a pairwise comparison of untreated and ICRF193-treated WT cells. Principal component analysis (PCA) clearly distinguished both groups of replicates (data not shown). DESeq2 differential expression analyses showed significant differences in the gene expression pattern of untreated versus ICRF193-treated WT cells, with 207 genes downregulated (fold change ≤ 0.5) and 197 genes upregulated (fold change ≥ 2) (Fig. 7A, see p-values in Table S1). The most significant downregulated genes were CEMIP (cell migration inducing hyaluronidase 1) (− 3.10 log2FC), KIF26B (kinesin family member 26B) (− 2.98 log2FC), and EXT1 (exostosin glycotransferase 1) (− 2.92 log2FC). Interestingly, CEMIP positively regulates epithelial-mesenchymal transition and therefore, tumor cell growth, invasion, and cancer dissemination^34,35^. CEMIP has been shown to regulate the WNT and EGFR signaling pathways which play a key role in embryonic development and cell proliferation^36^. Similarly, KIF26B is overexpressed in various cancers^37^, promoting cell cycle progression^38^ and regulating cell adhesion^39^, and EXT1 promotes cell proliferation and migration via WNT signaling pathway^40^. In this regard, the downregulation of these genes after ICRF193 treatment seems to accompany or maybe contribute to the cell cycle arrest induced by the activation of the DC checkpoint. The most significant upregulated genes were TINAGL1 (tubulointerstitial nephritis antigen-like 1) (4.03 log2FC), CCN5 (3.97 log2FC), and TPTEP2-CSNK1E (3.78 log2FC). On the opposite to the downregulated genes, TINAGL1 suppresses cancer progression and metastasis by inhibiting EGFR signaling^41^, reducing cell growth^42^. CCN5 (WNT1 inducible signaling pathway protein 2) inhibits cell proliferation and promotes apoptosis^43,44^. Interestingly, TOP2A mRNA levels were increased in response to ICRF193 (log2FC 1.27).

Next, we performed functional enrichment analyses for differentially expressed genes (absolute fold change ≥ 2) using Profiler in RStudio (Fig. 7B). The full list of terms with their corresponding p-values, intersection size, and enrichment ratio, can be found in Table S2. From the KEGG (Kyoto Encyclopedia of Genes and Genomes) database, we obtained sets of gene enrichment for several signaling pathways, among them (1) EGFR tyrosine kinase inhibitor resistance, (2) ErbB signaling pathway, (3) AGE-RAGE signaling pathway in diabetic complications, (4) Hippo signaling pathway, (5) Rap1 signaling pathway, (6) Cytokine-cytokine receptor interaction, (7) MAPK signaling pathway and (8) PI3K-Akt signaling pathway. The importance of these analyses lies in unveiling which signaling pathways are affected by prolonged catalytic inhibition of TOP2A activity and the prospective implications of this. In addition to studying cell cycle stage differences between control and treated samples.

Also of importance, we analyzed the REACTOME, identifying six pathways: (1) Neurophilin interactions with VEGF and VEGFR, (2) DNA Damage/telomere-induced senescence, (3) Transcriptional regulation of granulopoiesis, (4) PRC2 methylates histones and DNA, (5) Cell Cycle, Mitotic, and (6) signal transduction. Concerning the last two, since the cell cycle dynamic upon prolonged ICRF193 treatment is mainly altered at G2/M progression, the genes included in those terms might contribute to the pathway responding to the massive presence of chromatin-entrapped TOP2A closed-clamp complexes during the G2 phase of the cell cycle (see Table S3 for full description).

Last but not least, we compared the above-mentioned transcriptional profile of RPE1 WT cells upon prolonged ICRF193 incubation with the one observed in RPE1 MCPH1 KO cells after the same treatment. The objective of this analysis was to understand whether particular genes or expression patterns are relevant to confer adaptation ability to the G2-arrest caused by ICRF193 incubation since the arrest is bypassed in cells with functional MCPH1. To visualize the specific differences between RPE1 WT and MCPH1 KO upon ICRF193 incubation, the DEG data of both samples were normalized against each untreated counterpart. After performing this normalization under standard filtering conditions (i.e. adj.p.value ≤ 0.05 and absolute fold change > 2), we did not find upregulated or downregulated genes between WT and MCPH1 KO cells treated with ICRF193 (Fig. 7C). From these results, we can infer that global transcriptional profiles associated with TOP2A inhibition by prolonged ICRF193 treatment are not particularly affected by MCPH1 depletion, not showing differences concerning adaptation ability. In other words, the transcriptomic landscape rewires similarly in WT and MCPH1 KO cells upon prolonged exposure to ICRF193. In summary, the differences concerning the TOP2A-related G2 checkpoint adaptation capacity reported for WT cells do not show a specific transcriptional response reflected on abrupt changes in the mRNA levels for key regulatory genes. Therefore, it is likely that different cellular mechanisms, such as posttranscriptional events, protein–protein interactions, epigenetics changes, or the chromatin landscape, control the machinery of adaptation to the decatenation checkpoint.

Interestingly, a recent study has shown the novel role of H3K27 and H4K20 methylation in promoting the mitotic function of TOP2A to ensure faithful chromosome segregation^33^. This study suggests that the role of TOP2A chromatin tether domain is not to promote bulk decatenation of the genome, but to ensure the complete resolution of sister centromeres essential for high-fidelity chromosome segregation. Even small numbers of chromosome segregation errors or UFBs give rise to micronuclei that accumulate damaged DNA following cytokinesis^45^. Sister centromere catenations result in the accumulation of genomic lesions that contribute to tumorigenesis. In our previous work describing chromosomal structural deficiencies in MCPH1-depleted cells^25^, we showed the distribution of TOP2A in mitotic chromosomes. Compared with control, twisted chromosomes showed hypercoiled axes and diffused TOP2A signals in centromere regions. As shown in^33^ specific methyltransferase inhibitors reduce histone H3 or H4 methylation decreasing TOP2A at centromeres and increasing segregation errors. The phenotype these authors describe resembles our previous observations reporting diffused TOP2A signal in MCPH1 depleted chromosomes^25^, together with MCPH1 requirement for efficient chromosome alignment during mitosis. Whether and how the latter is related to DC adaptation and subsequent segregation errors (increase in the fraction of cells showing UFBs) needs to be further investigated. In this sense, it is tentative to speculate a connection between the MCPH1 and TOP2A-related phenotypes with H3/H4 methylation changes in these structural regions.

In the current literature, no direct connection has been made between the DC checkpoint and the above histone modifications. To date, H4K20 methylation has been shown to regulate replication licensing and has been linked to Meier-Gorlin Syndrome^46^, while H4K20me3 ensures timely heterochromatin replication by association with the DNA helicase^47^. However, it has been shown that the tumor suppressor SirT2 regulates cell cycle progression and genome stability by modulating the mitotic deposition of H4K20 methylation. SirT2 loss in mice induces significant defects associated with defective H4K20me1-3 levels, and accordingly, genomic instability and chromosomal aberrations related to tumorigenesis^48^. H4K20me1 were previously found to be tightly cell cycle-regulated suggesting that they play an important, although not clear role in cell cycle progression, affecting the re-replication of DNA and the improper timing of mitotic progression^49^. According to this evidence, the fine-tuned regulation and the genomic distribution of these histone modifications may play an important role in cell cycle progression and cell fate decisions. Whether they are affected by MCPH1 depletion in carcinogenic and/or non-carcinogenic models will be the subject of future research.

## Conclusions and outlook

In this study, we further dissected the adaptation pathway to the decatenation checkpoint activated by catalytic inhibition of TOP2A activity. In this regard, not much is known about molecular factors directly involved in the adaptation response, since it has been barely studied until recent years. Furthermore, inhibition of TOP2A activity has been mostly studied in cancer cells, due to its importance and use in cancer treatment. In a previous study, we have shown that downregulation of MCPH1 in HeLa cells negatively affected their adaptation to the decatenation checkpoint, increasing the robustness of the cell cycle arrest after MCPH1 depletion. We also showed that ICRF193-induced G2 arrest is not dependent on the p38 MAPK pathway in MCPH1-deficient cells^11^. In the adaptation signaling pathway, MCPH1 likely acts upstream of Plk1 by counteracting Chk1 activity to confer cellular adaptation to the decatenation checkpoint^12^. Here, we investigated the activation, robustness, and adaptation capability in a non-carcinogenic model, RPE1 cells. We found that MCPH1, previously identified as a key regulator of this checkpoint, affects the adaptation ability of the cells, and its depletion restores the robustness of the G2-arrest in a p53 deficient model. The data show that MCPH1 is a component of the decatenation checkpoint pathway in a non-carcinogenic model, being required for adaptation even in the absence of p53. In addition, by RNA-seq analysis we have shown that the adaptation pathway is not related to a clear transcriptional response. However, we did observe transcriptional changes related to catalytic inhibition of TOP2A.

Furthermore, we characterized the (patho-)physiological consequences in cells that bypass the decatenation checkpoint. There was a higher incidence of multilobulated cells with abnormal nuclear shapes and aberrant DNA content, in addition to anaphase bridges and UFBs. These defects could lead to genomic instability in the subsequent cell cycles and be related to higher apoptosis rates upon long-term catalytic inhibition of TOP2A. In this sense, MCPH1 boosts the occurrence of these phenotypes in WT cells, either as a direct consequence of checkpoint bypass with high levels of catenations, as a consequence of epigenetic differences and chromatin environment, or both. The latter makes MCPH1 an attractive target for cancer therapy in combination with the use of TOPO2 inhibitors, helping to improve the effectiveness of the treatments. A higher apoptosis rate in WT cells after TOP2A inhibition compared with MCPH1 KO, allows us to speculate that combined TOP2A inhibition and ectopic MCPH1 overexpression could hypothetically increase cell death due to rapid adaptation. This would especially affect carcinogenic cells. Different types of programmed cell death caused by the accumulation of reactive oxygen species hold the potential for the development of novel cancer therapies^50^. For example, the increase in reactive oxygen species by oxygen nanobubbles delivery using graphene/CuO_2_ nanoshuttles^51,52^. Their effects in mammalian carcinogenic cells, combined with other sources of genomic stress like TOP2A inhibition, may be a source of future research. Higher TOP2A expression has been related with proliferation and metastasis in kidney clear cell carcinoma, a type of cancer that exhibits a diverse therapeutic response to immune checkpoint inhibitors. In this context, TOP2A inhibition reduces cell growth and mobility, activating programmed cell death^53^. Interestingly, our study elucidates the effect of TOP2A-dependent cell cycle checkpoint adaptation on apoptosis, showing higher segregation defects and cell death for cells with functional MCPH1. The latter could be particularly interesting for cancer cells resistant to immune checkpoint inhibitors or those showing TOP2A-dependent checkpoint activation since controlling the adaptation response upon checkpoint activation can still activate programmed cell death.

Regarding genomic stability and tumorogenesis, several reports have shown that MCPH1 overexpression decreases cellular proliferation, and cell migration, and induces cell apoptosis, indicating its tumor suppressor activity^54^. Interestingly, a study has shown that overexpression of MCPH1 inhibits the migration and invasion of lung cancer cells by regulating proteins that control epithelia-mesenchymal transitions, among them p53^55^. MCPH1 is downregulated in tumor tissues and cancer cell lines and was considered a novel tumor suppressor gene^56^. Another study identifies MCPH1 deletion as a cause of centrosome amplification in human cancer^57^. Overall, the loss of MCPH1 expression plays a key role in promoting genome instability and mutations, supporting its function as a tumor suppressor gene. But, as frequently occurs in cancer studies, it is a complex scenario in which the same factor can play opposite roles depending on the specific molecular context. Therefore, identifying that “context” is specifically relevant for MCPH1 as a multifunctional protein. MCPH1-depleted cells, regardless of their defect on mitotic chromosome decatenation, showed different cell cycle dynamics and decatenation checkpoint responses in the presence of catalytic TOP2A inhibitors. This corresponds with a more stable cell cycle arrest that safeguards cells from going into mitosis with unresolved DNA catenations, reducing segregation defects and apoptosis. In this study, we have shown the effect of MCPH1 presence/absence in a p53-negative cell line, and how MPCH1 increases programmed cell death by increasing the leakiness of the decatenation checkpoint. The latter combination can be used to increase cell death of tumor cells. The next steps to study the potential of MCPH1 in cancer therapy should address the mapping and characterization of MPCH1 expression levels in several cancer types with different molecular backgrounds and their response to TOP2A catalytic inhibition.

In summary, by a combination of cell cycle profiling by flow cytometry, live-cell microscopy, and the transcriptomic analysis by RNA-seq, this study brings clarity into how cells respond to genomic stress caused by concatenation, including the consequences of adaptation to the checkpoint and its potential use in cancer therapy.

## Methods

### Cell culture, generation, and characterization of MCPH1 knockout cell line

hTERT RPE-1 cells were cultured in Dubelccos Modified Eagles Medium (DMEM) F-12 (Invitrogen) supplemented with 1 × penicillin/streptomycin and 10% Fetal Bovine Serum (FBS) at 37 ºC and 5% of CO2. As an MCPH1 mutated model, we made use of gene-edited cell clones obtained in the laboratory of Felipe Cortés-Ledesma (CNIO, Spain). To generate MCPH1 knockout cells, RPE1 TP53 −/− Cas9 cells (overexpressing Cas9)^58^ were cotransfected with the corresponding MCPH1-targeting gRNAs (5’ GGATGACCACACTTCAACAT) or non-targeting gRNA as a negative control and with Cas9-expressing construct using Lipofectamine RNAiMAX (Thermo Fisher) following the protocol provided by the manufacturer. Editing efficiency was validated by in-del analysis of PCR sanger sequencing using TIDE^59^. Upon selection of the obtained clones of interest, MCPH1-mutated clone #14 was selected for subsequent analyses as it included a 7 bp deletion in exon 2 which results in a truncated MCPH1 protein (c.MCPH1del79_85; p.V13Gfs*33). This protein domain is frequently mutated in human microcephaly patients^60,61^. The mutated allele was stable at mRNA as its levels were not found downregulated in RNAseq data analyses (see below). Inspection of seq reads by IGV confirmed that all MCPH1 transcripts from mutated cells included the 7 pb deletion (Fig. S1A). A similar maintained MCPH1 expression was previously described in a gene-edited breast epithelial cell model (MCF10A) including MCPH1 truncating mutations^62^. Our immunoblot analyses confirmed the absence in MCPH1 mutated RPE1 cells of both major protein isoforms coded by this gene, 110 kDa (full-length) and 75 kDa (∆9–14 variant) respectively (see Fig. S1B).

Of note, our RPE1 cell model was p53 mutant (c.332delT p.L111Rfs*122), since it was not possible to obtain viable RPE1 cells with CrisprCas-mutated MCPH1 under p53 WT background (this is likely explained by a synthetic viability phenomenon of interaction that is currently under investigation; F. Cortés-Ledesma personal communication). Before drug treatments, cells were seeded into fresh medium in 100 mm plates at 70% confluence one day before starting with drug incubations. Cells were treated with ICRF-193 (10 uM, SIGMA) or etoposide (10 uM, SIGMA) for the required time. Untreated cells were incubated with a similar volume of the solvent DMSO.

### Cytogenetic analyses

Cytogenetic preparations following standard protocols were obtained from synchronized cells at G1/S by double-thymidine blockage. Cells were processed 10–12 h after release from the second thymidine release to coincide with the mitotic wave. Nocodazole (Sigma-Aldrich, final concentration 1.5 uM) was added when indicated 10 h after the second thymidine release and maintained for 2 h before cell collection. Chromosome preparations were fixed using Carnoy’s solution (methanol or glacial acetic acid, 3:1), stained with Giemsa (10%), and finally visualized by bright-field microscopy. The fraction of prophase-like cells (PLCs) and metaphases was determined after counting 1000 nuclei from coded slides. Microscopy images were captured with a CCD camera (DP70; Olympus) coupled to a microscope (BX51; Olympus) and finally, managed with FiJi software.

### Western blot

Approximately 1 × 10^5^ cells were suspended in 100 μl of lysis buffer, sonicated, and boiled for 2 min. Proteins were resolved by SDS-PAGE and transferred to Hybond-P PVDF membranes (Amersham). The membrane was blocked with 2.5% (w/v) dry milk in TBS-T (20 mM Tris–HCl [pH 7.5], 150 mM NaCl, 0.05% Tween 20). Incubation with primary antibody (anti-MCPH1 ref.11962-1-AP, Proteintech; dilution 1:500) was performed in TBS-T containing 1% BSA and 0.05% sodium azide overnight at 4ºC. Anti-alpha tubulin (ref. T5168, Sigma; dilution 1:1000) was used as the loading control. Blots were developed by an enhanced chemiluminescence detection system (Amersham). Chemiluminescence signal in the membranes was developed in a dark room using a film system and a cassette, followed by immersion of the films in developing solutions. The membranes were cutted vertically prior to antibody incubation in order to optimize antibody usage. The films were cutted at the approximated size of the membranes, and the size of the bands was compared to the protein ladder in the membrane. Different exposure times in the cassette were applied for the films. After developing, the films were scanned and digitized. Full unprocessed scanned films for different exposition times are available in Supplementary data 1.

### Live-cell imaging

The procedure was similar to the one described in^11,17^. Cells were plated at 70% of confluence onto 35 mm four-square tissue culture dishes fitted with glass coverslips (MatTek Cultureware) and coated with gelatin. Double thymidine cell synchrony was performed as described in the results and discussion section. Upon release from the second thymidine incubation the standard medium containing the thymidine was exchanged for DMEM without phenol red, supplemented with 10% FBS, penicillin/streptomycin, 200 mM Trolox (Calbiochem), and 250 nM of SiR-DNA (Spirochrome) prewarmed in incubator at 37 ºC. When corresponding ICRF193 10 uM was also added. After 1 h of incubation, the medium was replaced by a fresh new one that did not contain SiR-DNA. The dishes were transferred to a microscope-humidified stage incubator containing 5% CO2 at 37ºC. Cells were filmed with three to five z sections using an inverted laser scanning Leica TCS SP5 microscope fitted with 20 × objective and zoom 2 × and coupled with Confocal LAS AF software (Leica Application Suite for Advanced Fluorescence). Cells were imaged live for 10 h after wash-out from SiR-DNA staining. TIFF images were stacked and processed using FiJi software. Timing data were obtained after visual inspection of a minimum of 50 cells. Statistical comparisons were done using Statgraphics software.

### Cell cycle profile and apoptosis detection by flow cytometry

For flow cytometry analyses, cells were seeded into fresh medium in 100 mm plates at 70% confluence one day before starting with drug incubations. Approximately one million cells were recovered, washed in PBS, and fixed in ice-cold Ethanol 70 overnight. Phospho-histone H3 positive cells were detected with a rabbit anti-histone H3PS10 antibody (Abcam, dilution of 1/250) and a donkey anti-mouse IgG FITC-conjugated secondary antibody (Santa Cruz). Propidium iodide (SIGMA) was used as a counterstain for DNA content. Fluorescence detection was performed using an analytical flow cytometer (LSR Fortessa, BD Bioscience) equipped with BD FACSDiva™ software for data acquisition. Quantitative cell cycle analysis was done with Flowing Software v.2.5.1 as explained in^17,24^.

For apoptosis detection, cells were processed with “Dead Cell Apoptosis Kit” (Invitrogen), which stains cells with Annexin V Alexa Fluor™ 488 and Propidium Iodide, following the manufacturer’s recommendations. Fluorescence reading was performed using an analytical flow cytometer (LSR Fortessa; BD Biosciences) equipped with BD FACSDiva software (BD Biosciences) for data acquisition. Data were visualized with Flowing Software v.2.5.1.

### Image analysis of nucleus circularity and DNA content

After the corresponding treatments, cells were fixed and DNA was stained with DAPI. Microscopy images were captured with a CCD camera (DP70; Olympus) coupled to a microscope (BX51; Olympus) and processed with FiJi. To further study the effect of TOP2A inhibition and checkpoint bypass on nucleus morphology (fraction of lobulated cells) and genomic stability (DNA content), different analyses were performed. The circularity of the nuclei was chosen as a shape descriptor and quantified for the different conditions using FiJi (https://fiji.sc/). Briefly, cell nuclei visualized with DAPI were segmented using the plugin StarDist, and further measurements of segmented nuclei provided individual values of circularity and DAPI sum intensity among others. Circularity measurements were plotted as a boxplot and DAPI sum intensity as a violin plot using RStudio (https://rstudio.com/).

### Immunofluorescence detection of Ultra Fine Bridges (UFBs)

Cells were seeded on gelatin-coated glass coverslips, previously sterilized with UV light. ICRF (4-[2-(3,5-Dioxo-1-piperazinyl)-1-methylpropyl]piperazine-2,6-dione) was added the next day and cells were processed 3 h after. Fixation was performed using a 4% solution of paraformaldehyde in 1X PBS (pH 7.4) for 10 min at room temperature. After washing off the PFA, the cells were permeabilized using 0,5% Triton in 1X PBS for 5 min at 4ºC. The cells were then blocked with a 3% BSA prepared in 1X PBS for 1 h. Following this, the cells were incubated with primary antibodies overnight (rabbit Anti-PICH #37, a gift from Y. Azuma, dilution 1/100; guinea pig anti-CENP-C) MBL_ref.PD030, dilution 1/1000). Next, cells were washed with 1X PBS for 10-minutes, thrice, and then treated with secondary antibodies for 1 h (mouse anti-rabbit GFP-conjugated dilution 1/200, Santa Cruz Biotechnology Sc-2359; goat anti-guinea pig TRITC-conjugated dilution 1/200, Abcam ab6905). After a new round of washes with 1X PBS, coverslips were mounted onto slide glasses with VECTASHIELD Antifade Mounting Medium containing DAPI (#H-1200, Vector Laboratory) and sealed using nail polish. For scoring the UFB phenotype, we focused on anaphase cells at a consistent stage by ensuring that the CENP-C signals between the segregating chromosomes were at a similar distance. All the UFBs that can be visualized were counted by switching the plane of imaging on a fluorescence microscope (BX51; Olympus). Representative images were captured with a charge-coupled device camera (DP70; Olympus) coupled to the microscope and finally managed with FiJi software. UFB data was obtained from a minimum of 100 cells visualized across three independent experiments with at least 30 cells being counted from each experiment. Statistical comparisons were done using Statgraphics software.

### RNA extraction, library preparation and RNAseq analysis

Total RNA was extracted using the RNAeasy kit (Qiagen, Germantown, USA, Cat.No.: 74104) following the manufacturer’s instructions. RNA samples were quantified by Qubit RNA BR Assay kit (Thermo Fisher Scientific) and the RNA integrity was estimated with Agilent RNA 6000 Nano Bioanalyzer 2100 Assay (Agilent). The RNA-Seq libraries were prepared with KAPA Stranded mRNA-Seq Illumina Platforms Kit (Roche) following the manufacturer’s recommendations. Briefly, 500 ng of total RNA underwent poly-A enrichment, fragmentation, and second-strand synthesis with dUTP. The resulting cDNA was adenylated, ligated with adaptors (Integrated DNA Technologies), and enriched with 15 PCR cycles. The size and quality of the libraries were assessed in a high-sensitivity DNA Bioanalyzer assay (Agilent). The libraries were sequenced on NovaSeq 6000 (Illumina) with a read length of 2 × 51 bp, following the manufacturer’s protocol for dual indexing. Image analysis, base calling, and quality scoring of the run were processed using the manufacturer’s software Real-Time Analysis (RTA 3.4.4). RNA-seq reads were mapped against the Homo sapiens reference genome (GRCh38) with STAR/2.7.8a^63^ using ENCODE parameters. Genes were quantified with RSEM/1.3.0^64^ with default parameters and using the human gencode.v42 annotation. Differential expression analysis was performed with the R package limma using Voom function^65^. Functional enrichment analysis of the differentially expressed genes was performed with g:Profiler^66^. IGV (v2.8.12) was used to visualize bam files of interest.

### Statistics and reproducibility

Data visualization and statistical analysis were performed using RStudio ((versions V1.2.5033 and V2023.03.1–446), https://rstudio.com/) and MicrosoftⓇ ExcelⓇ. Barplots show the average value of the distribution and the whiskers represent the standard deviation with a 95% confidence interval. Barplots show normalized averaged values, and error bars show the respective standard deviation. In all boxplots, the box represents 50% of the data, starting in the first quartile (25%) and ending in the third (75%). The line inside represents the median. The whiskers represent the upper and lower quartiles. Outliers are excluded and defined as 1.5 times the interquartile range. The violin plots depict the density curves of the numeric data. The width of each curve corresponds with the approximate frequency of data points in each region. In the middle of each density curve is a small box plot, with the rectangle showing the ends of the first and third quartiles and the central dot the median. For the statistics, an independent two-group comparison was made for all conditions with the Wilcoxon-Mann–Whitney test (using default parameters, non-parametric, dependent variable). Related to this, n.s., not significant, is given for p-values > or equal to 0.05. The p-values are given in the plots between the top of two boxes subjected to comparison. For each experiment, two replicates were performed unleash stated otherwise in the figure legends.

## Supplementary Information

Supplementary Information 1.

Supplementary Information 2.

Supplementary Information 3.

Supplementary Information 4.

Supplementary Information 5.

## Data Availability

The data supporting this study are available from the corresponding author upon request. The RNAseq datasets generated and analysed during the current study are available in Sequence Reads Archive (SRA), with the accession number GSE286076, project PRJNA1205784, available at https://dataview.ncbi.nlm.nih.gov/object/PRJNA1205784?reviewer=gp4m0cvk3hj0o44nmet9rl3u0m.
