# Author Correction: TOP2A inhibition and its cellular effects related to cell cycle checkpoint adaptation pathway

**DOI:** 10.1038/s41598-025-97106-z

**Published:** 2025-04-11

**Authors:** Maria Arroyo, M. A. Fernández-Mimbrera, E. Gollini, A. Esteve-Codina, A. Sánchez, Juan Alberto Marchal

**Affiliations:** 1 Departamento Biología Experimental, Universidad de Jaén, Paraje Las Lagunillas S/N E23071, Jaén, Spain; 2 Centre Nacional d’Anàlisi Genòmica (CNAG), Baldiri Reixac 4, 08028 Barcelona, Spain; 3 Universitat de Barcelona (UB), Barcelona, Spain; 4 Cell Biology and Epigenetics, Department of Biology, Technical University of Darmstadt, Darmstadt, Germany

Correction to: *Scientific Reports* 10.1038/s41598-025-87895-8, published online 30 January 2025

The original version of this Article contained an error in the spelling of the author Maria Arroyo and Juan Alberto Marchal which were incorrectly given as Maria C. Arroyo López and Juan Alberto Marchal Ortega.

The original Article has been corrected.

