# Supplementary material for: TOP2A inhibition and its cellular effects related to cell cycle checkpoint adaptation pathway
Source: Sci Rep. 2025 Jan 30;15:3831. doi: 10.1038/s41598-025-87895-8 (PMC11782647; doi:10.1038/s41598-025-87895-8)
Supplement: Supplementary file 1 — Supplementary Information 1. [file 41598_2025_87895_MOESM1_ESM.pdf]

SUPPLEMENTARY MATERIAL

TOP2A inhibition and its cellular effects related to cell cycle checkpoint adaptation pathway

AUTHORS

Arroyo M*[#4], Fernández-Mimbrera MA[#1], Gollini E[1], Esteve-Codina A[2,3], Sánchez A[1], Marchal JA*[1].

AFFILIATIONS

1) Departamento Biología Experimental, Universidad de Jaén, Paraje Las Lagunillas s/n E23071, Jaén, Spain

2) Centre Nacional d'Anàlisi Genòmica (CNAG), Baldiri Reixac 4, 08028 Barcelona, Spain

3) Universitat de Barcelona (UB), Barcelona, Spain

4) Cell Biology and Epigenetics, Department of Biology, Technische Universität Darmstadt, Germany

# These authors contribute equally to this work.

* Corresponding authors:

Prof. Dr. Juan Alberto Marchal Ortega. Tel: 0034-953213361; Email: jamaor@ujaen.es

Dr. Maria C Arroyo López. Email: arroyo.lopez.mc@gmail.com

SUPPLEMENTARY FIGURES

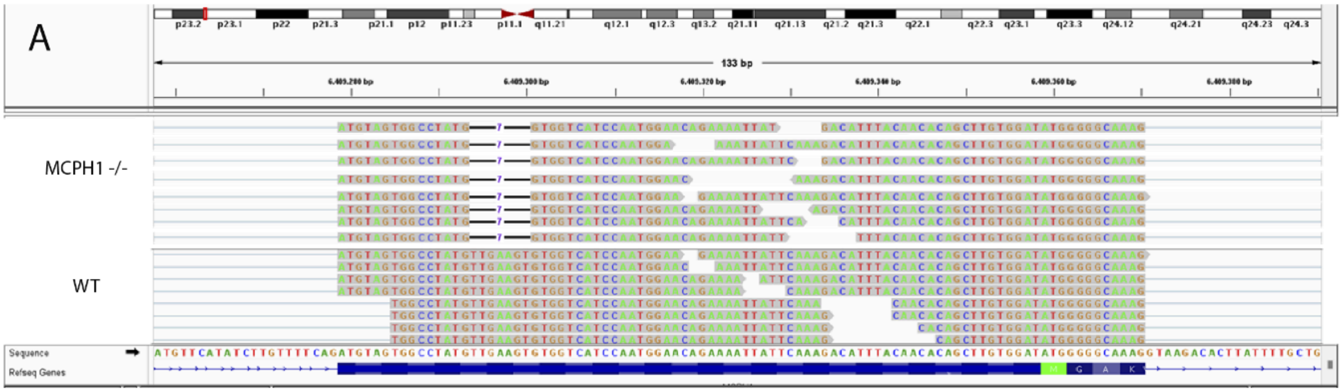

IGV visualization of seq reads confirmed that all MCPH1 transcripts from mutated cells included a 7 pb deletion in exon 2 (c.MCPH1del79_85; p.V13Gfs*33)

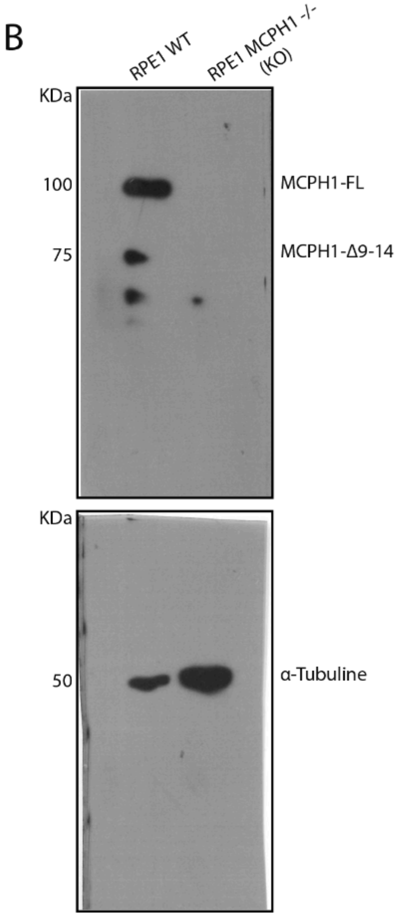

Figure S1. Characterization of RPE1 MCPH1 -/- knockout cell line. (A) On the top, is the scheme of the genomic locus for MCPH1 (reference genome GRCh38, chromosome 8). Genomic editing efficiency was validated by in-del analysis of PCR sanger sequencing. The alignment of sequences for MCPH1 KO cell lines versus wild-type shows the deletion of 7 pb in exon 2. (B) Western blot analysis of MCPH1 levels in RPE1 WT versus MCPH1 KO. The scan of the membrane shows the knockout of both MCPH1 isoforms in genetically edited RPE1 cells, 110 kDa (full-length) and 75 kDa (Δ9-14 variant) respectively.

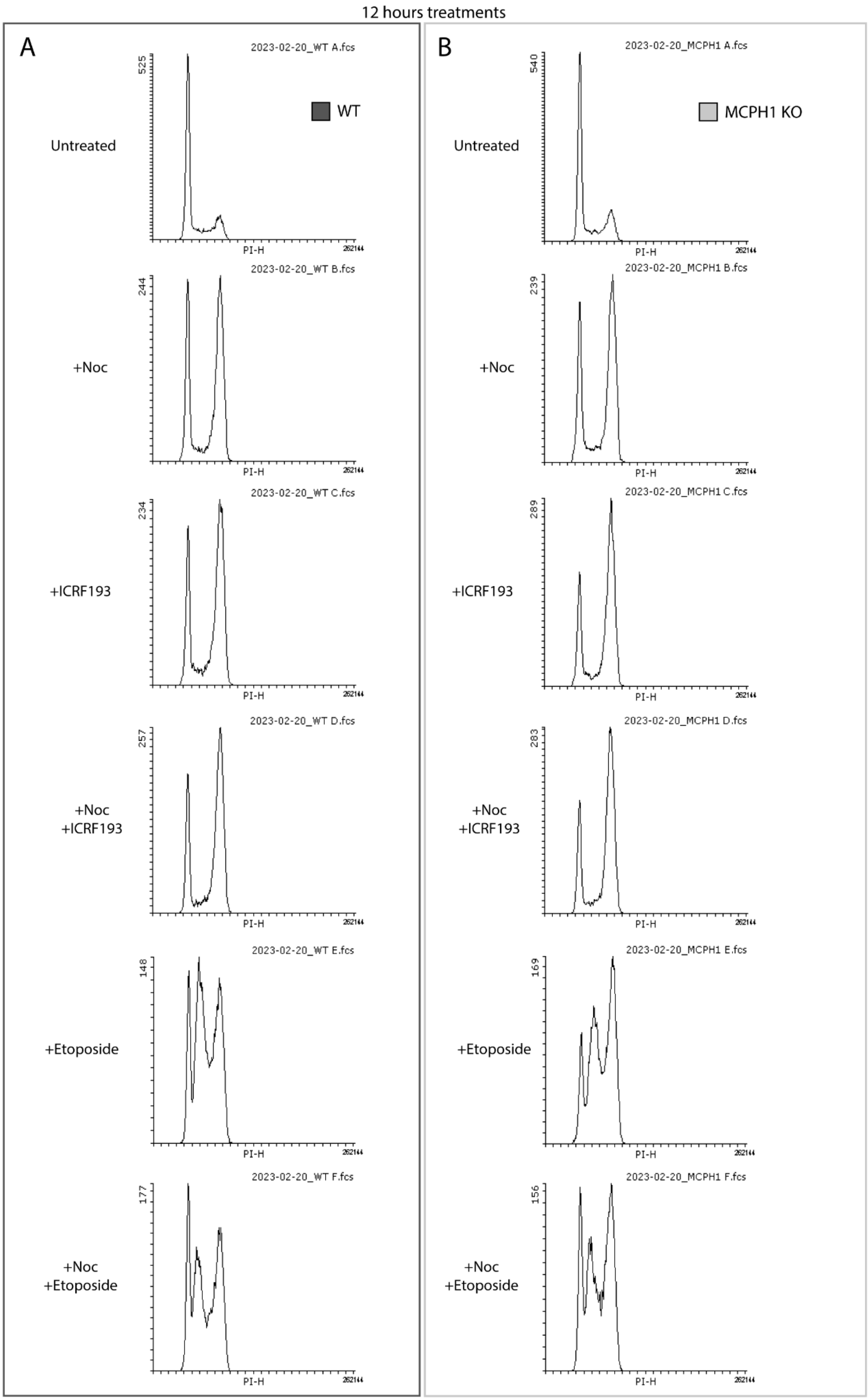

12 hours treatments
A
2023-02-20_WT A.fcs
WT
Untreated
+Noc
+ICRF193
+Noc
+ICRF193
+Etoposide
+Noc
+Etoposide
2023-02-20_WT B.fcs
2023-02-20_WT C.fcs
2023-02-20_WT D.fcs
2023-02-20_WT E.fcs
2023-02-20_WT F.fcs
525
244
234
257
148
177
PI-H
B
2023-02-20_MCPH1 A.fcs
MCPH1 KO
2023-02-20_MCPH1 B.fcs
2023-02-20_MCPH1 C.fcs
2023-02-20_MCPH1 D.fcs
2023-02-20_MCPH1 E.fcs
2023-02-20_MCPH1 F.fcs
540
239
289
283
169
156

Figure S2. Histograms showing propidium iodide (PI) profiles obtained by flow cytometry analysis after 12 hours of treatments. Untreated (Control), Nocodazole (+Noc), +Noc+ICRF193, +Etoposide, +Noc+Etoposide. (A) Wild-type cells (WT). (B) MCPH1-depleted cells.

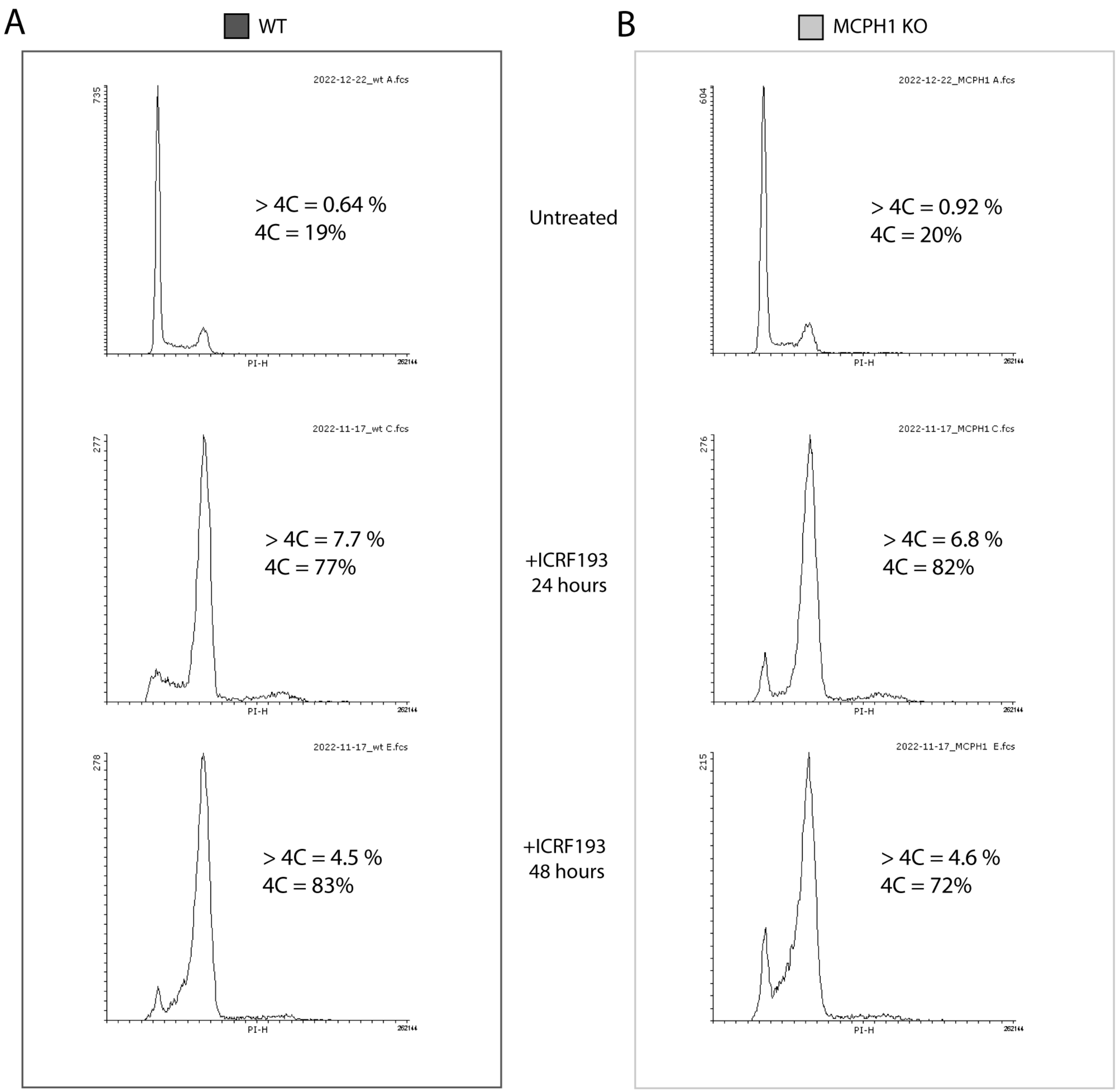

Figure S3. Histograms of propidium iodide (PI) profiles obtained by flow cytometry analysis after 24 and 48 hours of treatments. Untreated (control), +ICRF193. (A) Wild-type cells (WT). (B) MCPH1-depleted cells. The percentage of cells with a c-value (DNA content) bigger than, or equal to 4C is shown for each condition.
