# Supplementary figures and images for: TOP2A inhibition and its cellular effects related to cell cycle checkpoint adaptation pathway
Source: Sci Rep. 2025 Jan 30;15:3831. doi: 10.1038/s41598-025-87895-8 (PMC11782647; doi:10.1038/s41598-025-87895-8)

Unprocessed scanned films from Figure S1B:

anti-MCPH1

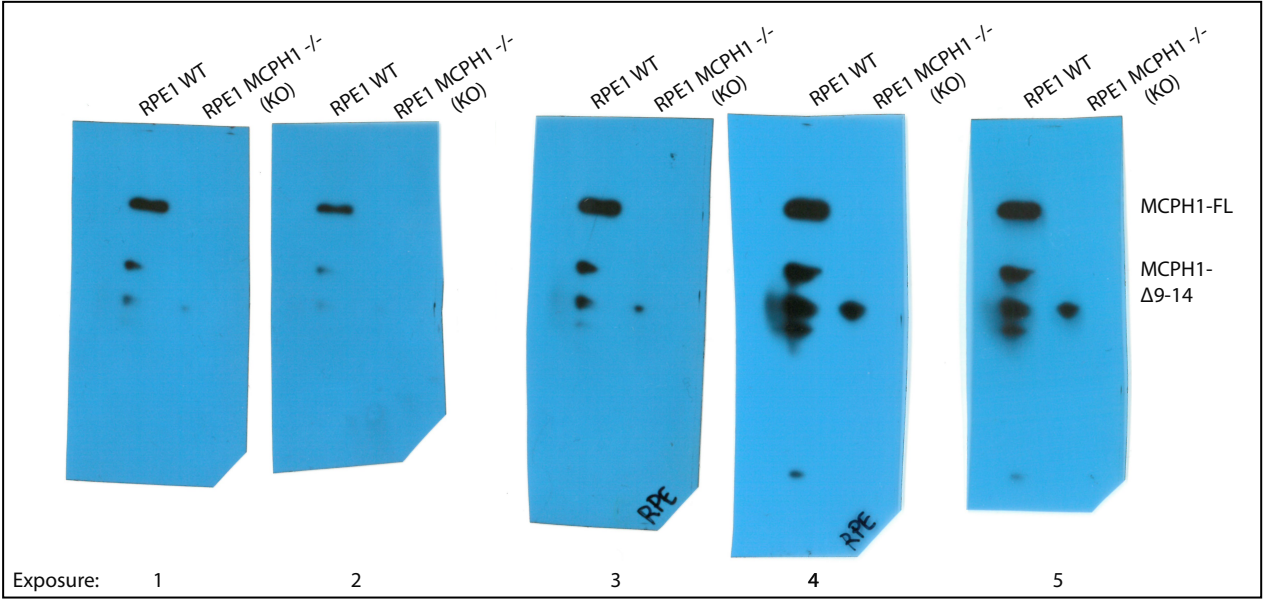

anti-Tubuline

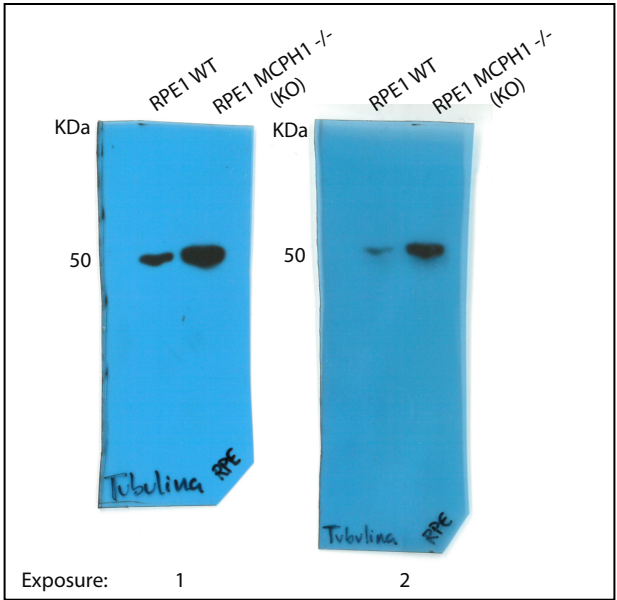

Supplement: Supplementary file 5 — Supplementary Information 5. [file 41598_2025_87895_MOESM5_ESM.pdf]
